# Human primordial germ cell heterogeneity in vitro is associated with distinctive biological states

**DOI:** 10.1186/s11658-026-00986-w

**Published:** 2026-06-24

**Authors:** Young Sun Hwang, Qiu Ya Wu, Sissy E. Wamaitha, Amander T. Clark

**Affiliations:** Department of Molecular, Cell, and Developmental Biology, Eli and Edyth Broad Center for Regenerative Medicine and Stem Cell Research, Center for Reproductive Science, Health and Education, University of California, Los Angeles (UCLA), Los Angeles, 90095 CA USA

**Keywords:** Human in vitro germ cells, Heterogeneity, Extended culture, Dead end 1, Cell cycle exit, Post-transcriptional regulation

## Abstract

**Background:**

Primordial germ cells (PGCs) exhibit molecular heterogeneity during development, yet whether this reflects functionally distinct states remains unclear.

**Methods:**

We investigated the functional significance of DND1 translation heterogeneity in human PGC-like cell (hPGCLC) extended culture, using dual fluorescent reporters, transcriptomic analysis and functional assays.

**Results:**

Single-cell RNA-sequencing revealed that DND1 + hPGCLCs are enriched in RNA regulatory genes including those associated with P-bodies and translational control. DND1 + cells exhibit reduced proliferation, cell cycle exit, and elevated nuclear expression P27 (CDKN1B). These cells also maintained stable expression of genes essential for germ cell differentiation. Notably, BMP2 supplementation stabilized DND1 translation without altering mRNA levels or proliferation rates.

**Conclusions:**

Together, these findings demonstrate that post-transcriptional regulation by DND1 coordinates cell cycle dynamics with distinct cellular state in human germ cells, highlighting critical translational control mechanisms in germline development.

**Supplementary Information:**

The online version contains supplementary material available at https://doi.org/10.1186/s11658-026-00986-w.

## Background

Primordial germ cells (PGCs) constitute the founding cellular population of the germline, the sole lineage through which genetic and epigenetic information is passed between generations to ensure species continuity. PGCs emerge during early embryonic development and ultimately differentiate into gametes, eggs or sperm, following a carefully orchestrated developmental program characterized by unique epigenetic and genetic reprogramming, including global DNA demethylation, chromatin remodeling and meiosis [1–4]. Defects in germ cell development contribute to infertility and can lead to germ cell tumors such as seminomas and testicular teratomas [5–7]. These are diseases of significant public health relevance as infertility affects approximately 1 in 6 individuals of reproductive age across their lifetime [8] and testicular cancer will affect around 1 in 250 men at some point during their lifetime [9]. Therefore, understanding the molecular mechanisms governing PGC specification, migration, and differentiation is critical for reproductive medicine, assisted reproductive technologies, and cancer biology.

In mammals, PGC specification occurs through inductive signaling from surrounding tissues rather than through inheritance of preformed germ plasm [10, 11]. In mice, PGCs are specified around embryonic day 6.5 (E6.5) through BMP4 signaling to WNT3-primed epiblast cells, activating a core transcriptional network comprising Blimp1 (Prdm1), Prdm14, and Tfap2c [12–14]. Human PGC specification shares some aspects with the mouse while also exhibiting fundamental differences. For example, specification in humans depends on SOX17 and TFAP2C which acts upstream of BLIMP1 and PRDM14 [15–18]. Following specification, mouse PGCs undergo extensive migration and proliferation while initiating genome-wide epigenetic reprogramming [19, 20]. During the initial post-specification period (E7.75-E9.5), mouse PGCs exhibit unique cell cycle dynamics with G2 phase arrest and transcriptional quiescence, concomitant with dramatic changes in histone modifications including loss of H3K9me2 and gain of H3K27me3 [3, 20]. After E9.75, PGCs resume rapid proliferation as they colonize and settle within the genital ridges at E10.5-E11.5, while continuing their epigenetic remodeling. From E11.5-E13.5 PGCs complete erasure of imprinted genes, undergo dynamic changes in TE expression and initiate sex-specific differentiation [20–24].

In humans, PGCs are first located in the yolk sac at 3–4 weeks post-conception (wpc). From there, they travel to the hindgut epithelium, where they are hypothesized to migrate along nerve fibers through the dorsal mesentery, to reach the genital ridge at around 5 wpc. During this journey PGCs undergo epigenetic reprogramming [25–28] and are likely proliferative given that approximately 3,000 human PGCs can be identified at 5 wpc [29]. While the precise cell cycle dynamics associated with human PGC specification and migration remain incompletely characterized, once in the gonad, PGCs undergo rapid proliferation and continue epigenetic reprogramming before initiating sex-specific differentiation from 8–9 wpc [1, 28, 30, 31]. The conservation of epigenetic reprogramming and proliferation patterns are shared features of mouse and human PGC development suggests a putative quality control checkpoint allowing epigenetic and genetic surveillance, or a strategy to prevent premature differentiation into somatic lineages [19, 32–34]. Single-cell analyses have revealed subtle yet important molecular heterogeneity within the human PGC population, challenging the traditional view of PGCs as a uniform homogenous cell type [1, 30, 35]. This heterogeneity manifests at a molecular level through the differential expression of pluripotent transcription factors, germ cell-expressed genes, metabolic genes related to oxidative phosphorylation, and regulators of growth and translation, such as components of the MYC pathway, ribosomal proteins and translation machinery. Collectively, this suggests that within the PGC population, germ cells can co-exist in a variety of closely related developmental states [30, 35, 36]. It is speculated that these different states endow PGCs with distinct biological properties.

Dead end 1 (DND1), an evolutionarily conserved RNA binding protein (RBP) is essential for PGC survival and tumor suppression in mice and zebrafish. Dnd1 is first expressed in mouse PGCs at the time of PGC specification at E7.5 [37, 38]. In humans, *DND1* RNA is first detected at 2–3 wpc [39]. In mice, a point mutation in *Dnd1* is the genetic cause of the *Ter* mutation, which universally results in germ cell loss when bred onto different mouse genetic backgrounds, and is a modifier of testicular germ cell tumor incidence on the 129 strain of inbred mice [38]. Unlike mice, germline mutations in *DND1* do not appear to have a major role in human testicular germ cell tumor incidence [40, 41]. Yet its role in other aspects of human PGC biology remains unexplored. Mechanistically in somatic cells, DND1 regulates post-transcriptional gene expression through protecting certain mRNAs from miRNA-mediated repression while recruiting the CCR4-NOT deadenylase complex to destabilize others [42–44]. Emerging evidence suggests these DND1-dependent mechanisms may contribute to germ cell heterogeneity. For example, Dnd1-high male germ cells during late gestation in mice display elevated expression of epigenetic regulators and translational machinery compared to Dnd1-low cells [36]. However, the biological significance of this molecular heterogeneity, and whether it represents asynchronous developmental progression, functionally distinct cell states, or differential physiology, remains poorly understood, particularly in humans.

The scarcity of human embryonic material makes systematic studies of post-specification and pre-gonadal stage human PGC biology exceptionally challenging [45]. In vitro gametogenesis (IVG) from pluripotent stem cells provides a model for elucidating mechanisms of germ cell specification and pre-gonadal development using human cells. [46, 47]. Several groups have established robust protocols for deriving in vitro human germ cells called PGC-like cells (hPGCLCs) in vitro [16, 17], which differentiate further into either oogonia-like cells or pro-spermatogonia by aggregate culture with xenogenic somatic cells or co-cultured with hindgut organoids [48–50]. Extended culture systems can also model post-specification stages, specifically corresponding to the 2–3 weeks between PGC specification and gonadal colonization in vivo [51–53]. Using the in vitro differentiation platform to generate hPGLCLCs, we used single cell RNA-Seq to explore the heterogeneity of post-specified PGCLCs leading to the discovery that *DND1* RNA discriminates a unique hPGCLC subtype which is enriched in regulators of RNA biology. Utilizing reporter tools to discriminate between DND1 + and DND1- PGCLCs, our work provids unique biological insights into these two highly related germ cell subpopulations which co-exist in the same in vitro platform despite their unique and quantifiable biological differences.

## Methods

### hESC (human embryonic stem cell) cultures

UCLA2 hESCs [54] were maintained feeder-free on iMatrix-511 Silk (Amsbio, 892,021)-coated plates in StemFit^®^ Basic03 (Ajinomoto, SFB-503) supplemented with Recombinant Human FGF basic (Peprotech, 100-18B) at 37 °C in 5% CO_2_. For passaging, cells were dissociated by a 15-min incubation at 37 °C in a 1:1 combination of 0.5 mM EDTA/PBS and TrypLE Select (Gibco, 12,563,011), yielding a final 0.5 × TrypLE Select solution. Dissociated cells were seeded onto 6-well plates (Corning, 3516) at 2 × 10^3^ cells/cm^2^ in medium supplemented with 10 µM Y-27632 ROCK inhibitor (Tocris, 1254) on the first day post-passage. Mycoplasma testing was conducted routinely using the MycoAlert Detection Kit (Lonza, LT07-318), and all lines were confirmed negative.

### Generation of hESC reporter line

Donor vectors for CRISPR-mediated knock-in were constructed as follows. For the *TFAP2C-p2A-eGFP* (AG) allele, left (1147 bp) and right (1130 bp) homology arms were PCR-amplified from UCLA2 hESC genomic DNA and cloned into pCR-Blunt II-TOPO (Thermo Fisher Scientific, K280020SC). A p2A-eGFP cassette together with a loxP-flanked PGK-Puro selection cassette was introduced in-frame at the *TFAP2C* 3′ coding end by NEBuilder^®^ HiFi DNA Assembly Master Mix (New England Biolabs, E2621S), with the native stop codon removed to enable in-frame p2A-EGFP translation. Guide RNAs targeting PAM sites flanking the TFAP2C 3′ coding region (sgRNA sequences: AGCTGATTCTAACAAAACCC and TCAGCTGGACTCTGGTCTCC) were designed using the Benchling CRISPR tool and introduced into the pX335-U6-Chimeric BB-CBh-hSpCas9n (D10A) vector (Addgene, #42,335) [55]. An analogous strategy was applied for the*DND1-p2A-tdTomato* (DT) allele, using homology arms of 1,500 bp (left) and 921 bp (right). A loxP-flanked PGK-Neo cassette was incorporated alongside p2A-tdTomato at the *DND1* 3′ coding end, with sgRNAs TGACTCCATTCTTTCTCCAC and ATGGAGTCACTGTTTAACCA directing Cas9n to the target site.

For AG reporter establishment, 8 × 10^5^ UCLA2 hESCs received the AG donor vector (2.5 µg) alongside equal amounts of each sgRNA/nCas9 vector (1.25 µg each) via nucleofection with a P3 Primary Cell 4D X Kit L (Lonza, V4XP-3024). Puromycin selection (0.3 µg/mL) was used to isolate single colonies, from which monoallelic AG lines were identified and carried forward. The AGDT dual-reporter line was derived by introducing the DT donor and sgRNA/nCas9 vectors into UCLA2 AG hESCs, followed by neomycin selection (150 µg/mL). To remove antibiotic resistance cassettes, neomycin-resistant cells received 2.5 µg of a Cre recombinase-expressing plasmid, excising both PGK-Puro and PGK-Neo. Successful targeting and Cre-mediated recombination were verified by colony PCR, and the absence of random integration was confirmed in parallel. Karyotypic normality of all lines was established by G-banding analysis at Cell Line Genetics (Madison, WI), with clone 16 selected as the AGDT line (RRID: CVCL_F1HB) for all downstream experiments.

### hPGCLC induction

hPGCLC induction followed established iMeLC-intermediate protocols [17] with the following conditions. To generate iMeLCs, hESCs were seeded at 200,000 cells/well on 12-well plates coated with human fibronectin (Millipore, FC010) in GK15, composed of GMEM (Gibco, 11,710–035) with 15% KSR (Gibco, 10,828–028), 1 × NEAA (Gibco, 11,140–050), 1 mM sodium pyruvate (Gibco, 11,360–070), and 0.1 mM 2-mercaptoethanol (Gibco, 21,985–023), supplemented with 50 ng/mL Activin A (Peprotech, AF-120-14E), 3 µM CHIR99021 (Reprocell, 040004), and 10 µM Y-27632. Following a 48-h induction, iMeLCs were dissociated to single cells using 0.5 × TrypLE Select and transferred to low-adhesion V-bottom 96-well plates (Greiner, 651,970) at 3,500 cells/well in GK15 containing 200 ng/mL BMP4 (R&D Systems, 314-BP),, 1,000 U/mL LIF (Millipore, LIF1010), 50 ng/mL EGF (R&D Systems, 236-EG), 100 ng/mL SCF (R&D Systems, 255-SC), and 10 µM Y-27632.

### hPGCLC extended culture

Extended hPGCLC culture followed Gell et al. [51] with minor modifications. STO feeder cells (ATCC, 1503) were propagated in DMEM high glucose (Gibco, 11,960–069) supplemented with 10% FBS (Gibco, 26,140–079), 1 × penicillin–streptomycin/glutamine (Gibco, 10,378–016), and 50 ng/mL primocin (Invivogen, ANTPM2). Prior to use as feeders, STO cells were mitotically inactivated by Mitomycin C (MMC; Sigma, M0503) treatment at 10 µg/mL for 3 h, then collected with 0.25% Trypsin/EDTA (Gibco, 25,200–056). hPGCLCs were established on MMC-treated STO monolayers (MMC-STO) at 3,000–5,000 cells/well in 24-well plates pre-seeded with 200,000 STOs/cm^2^, in 1 mL FR10 medium (GMEM containing 10% KSR, 2.5% FBS, 1 × NEAA, 1 mM sodium pyruvate, 0.1 mM 2-mercaptoethanol, 1 × penicillin–streptomycin/glutamine, 100 ng/mL SCF, 10 µM forskolin [Sigma, F6886], and 10 µM rolipram [Sigma, R6520]) with 10 µM Y-27632 on day 0. Full medium exchange was performed at day 2, followed by 50% medium replacement every other day thereafter. Cultures were passaged every 10–14 days by FACS (BD FACSAria). For D4C21 cultures, MMC-STO density was increased by supplementing with half the original cell number at day 10. BMP2-supplemented cultures followed Murase et al. [53] with minor modifications: hPGCLCs were maintained on MMC-treated m220 feeders (2 µg/mL MMC for 2 h) in aRK15 medium (15% KSR, 2.5% bovine serum, 1 × GlutaMAX [Gibco, 32,571–036], 1 × penicillin–streptomycin, 0.1 mM 2-mercaptoethanol, 100 ng/mL SCF, 10 µM forskolin, 20 ng/mL bFGF, and 1.5 µM endo-IWR1 [Tocris, 3532]) supplemented with 100 ng/mL BMP2 (R&D Systems, 355-BM-010/CF).

### Fluorescence-activated cell sorting (FACS)

Floating aggregates or extended culture hPGCLCs were incubated in 0.1% Trypsin/EDTA (Gibco, 15,400–054) at 37 °C for 15 min with intermittent pipetting. Dissociation was stopped by adding an equal volume of either FBS (for aggregates) or FR10 medium containing RQ1 RNase-Free DNase (Promega, M6101) and 10 µM Y-27632 (for extended cultures). Cells were then resuspended in 0.1% BSA in DPBS and passed through tubes capped with cell strainer caps (Falcon, 352,235) prior to sorting or analysis. Preparative sorting was performed on a BD FACSAria, with sorted populations collected directly into FR10 medium. Analytical flow cytometry used a FACSFortessa (BD Biosciences). Data acquisition used FACSDiva Software (BD Biosciences) and all downstream analysis was carried out in FlowJo v10.10.0 (BD Biosciences).

### Single cell RNA-seq (scRNA-seq)

scRNA-seq libraries were generated from FACS-sorted hPGCLCs using the Chromium Single Cell 3′ v2 platform (10X Genomics). Briefly, single cells were encapsulated with barcoded gel beads to form GEMs (gel beads-in-emulsions), within which mRNAs were reverse-transcribed and the resulting cDNAs amplified. Sequencing-ready libraries were constructed from the amplified material and run on a NovaSeq S4 flowcell (custom PE100). Demultiplexed FASTQ files were processed with Cell Ranger v8.0 (10X Genomics), which performed alignment to GRCh38 and generated feature-barcode count matrices. Per-cell quality metrics, including unique feature count (nFeature_RNA), total UMI count (nCount_RNA), and mitochondrial transcript fraction, were computed for downstream filtering.

Seurat v5.0 [56] in R v4.5.1 was used for all single-cell analysis. Low-quality cells were excluded using thresholds of 200–7,500 unique features and mitochondrial gene fraction below 10%. Both replicates were integrated with the merge function, assigning unique cell ID prefixes to preserve replicate labels. Expression values were log-normalized (scale factor: 10,000), and the 2,000 most variable features were selected by variance-stabilizing transformation (VST) for use in downstream dimensionality reduction. Scaled expression values were computed across all genes via ScaleData. PCA was applied to highly variable features, with the appropriate PC number determined from elbow plots; UMAP embedding used the top 20 PCs. Neighbor graphs were built from the top 10 PCs using FindNeighbors, and unsupervised clustering at resolution 0.1 was performed with FindClusters. Cluster marker genes were called by FindAllMarkers, requiring expression in ≥ 25% of cluster cells and a minimum log fold-change of 0.25, with Bonferroni-adjusted *p*-values. Functional enrichment of marker gene sets was performed via g:Profiler [57], querying GO biological processes, molecular functions and cellular components, KEGG pathways, and Reactome pathways under default settings with Benjamini–Hochberg correction; terms reaching adjusted *p* < 0.05 were retained. All analyses used Seurat, dplyr, ggplot2, patchwork, viridis, and cowplot.

### Immunofluorescence

hPGCLC cultures were fixed with 4% paraformaldehyde, diluted from 16% Formaldehyde, Methanol-free (Thermo Fisher Scientific, 28,908) in 1 × PBS, for 15 min at room temperature, then permeabilized for 30 min on a shaker in PBST (0.1% Tween-20 [Fisher Scientific, BP337-100] in PBS) containing 0.1% sodium dodecyl sulfate (Fisher Scientific, AAJ60015AC). Blocking was carried out in 5% Normal Donkey Serum (NDS), Unconjugated (Jackson Immuno Research Labs, 017000121) in PBST for 1 h at room temperature. Primary antibodies diluted in in 5% NDS/PBST were applied overnight at 4 °C on a shaker; full antibody details are provided in Table S2. Secondary detection used donkey-raised antibodies at 1:500 dilution together with 1 µg/mL DAPI (Thermo Fisher Scientific, D1306) in 5% NDS/PBST for 50 min at room temperature. All imaging was conducted in 24-well 1.5H glass bottom black culture plates (0.170 ± 0.005 mm; Cellvis, P24-1.5H-N) on a Zeiss LSM 880 confocal laser-scanning microscope with Zeiss Zen 3.7 software, using Plan-Apochromat 20 ×/0.8 NA and 40 ×/1.4 NA M27 objectives. MKI67 signal quantification was performed in FIJI (ImageJ, NIH) [58].

### Cell cycle analysis by EdU and DAPI incorporation

To assess S-phase incorporation, a 10 mM stock of EdU was diluted in culture medium to a working concentration of 10 µM. Then, the complete medium in the hPGCLC cultures was replaced with the EdU-containing medium. After a 90-min pulse at 37 °C in 5% CO_2_, cells were dissociated and sorted on a BD FACSAria. EdU detection was performed using the Click-iT Plus EdU Alexa Fluor 647 Flow Cytometry Assay Kit (Invitrogen, C10635) per the manufacturer's protocol. Sorted, labeled cells were counterstained with 1 µg/mL DAPI and analyzed on a FACSFortessa.

### RT-qPCR

Total RNA was isolated from hPGCLCs using the RNeasy Micro Kit (Qiagen, 74,004) following the manufacturer's protocol, with RNA concentration measured on a Qubit 4 fluorometer (Invitrogen). First-strand cDNA was synthesized from 1 ng total RNA using the Maxima First Strand cDNA Synthesis Kit for RT-qPCR (Thermo Scientific, K1641). Quantitative PCR was run on a CFX Connect Real-Time System (Bio-Rad) with TaqMan™ Universal Master Mix II, with UNG (Applied Biosystems, 4,440,038) and gene-specific TaqMan primers (Table S3). Relative expression was calculated as *∆C*_*t*_ normalized to the mean of *ACTB* and *GAPDH* reference genes and reported on a log₂ scale.

### Bulk RNA-seq

Total RNA from sorted and pelleted cells was isolated using the RNeasy Micro Kit (Qiagen). cDNA libraries were constructed with the Ovation RNA-seq System V2 (Tecan Genomics, 7102) and cleaned using Beckman Coulter Agencourt beads (Beckman Coulter, A63881). SPIA-amplified cDNA was further purified through a Qiagen MinElute column (Qiagen, 28,206) and eluted in 50 µL low-EDTA TE buffer, then mechanically sheared to ~ 200 bp fragments on a Covaris S2 sonicator (Covaris, Woburn, MA). Sheared material was re-purified on a MinElute column and eluted in 10 µL, after which 8 µL was end-repaired and indexed using the Ovation Ultralow System V2 (Tecan Genomics, 0344). Final library cleanup used Agencourt XP beads; sequencing was performed on an Illumina NovaSeq X Plus at 2 × 100 bp.

Read quality was assessed with FastQC v0.11.2. Trimmed reads were aligned to hg38 with the Rsubread aligner v1.24.1 [59], and per-gene fragment counts summarized with featureCounts [60] using the Rsubread built-in hg38 annotation. DESeq2 identified differentially expressed genes at FDR ≤ 0.05. GO term enrichment used gProfiler [57] against a *Homo sapiens* background, retaining terms with adjusted *p* < 0.05.

### Whole-genome bisulfite sequencing (WGBS)

gDNA was extracted from FACS-isolated cells using the Quick-DNA Miniprep Kit (Zymo Research, D3020). Each 1 ng gDNA input was taken through bisulfite conversion and library construction with the Pico Methyl-Seq Library Prep Kit (Zymo Research, D5455) per the manufacturer's protocol. Library quality, fragment size, and molarity were assessed on a TapeStation 4200 (Agilent D1000 ScreenTape, 50,675,582), and all libraries were sequenced on an Illumina NovaSeq X Plus with the 150-cycle kit.

Sequencing read quality was evaluated with FastQC. Adapter sequences were removed using Cutadapt, retaining read pairs ≥ 30 bp, and trimmed reads were aligned to the hg38 reference (3-letter conversion) with bsbolt Align under default parameters. CpG methylation was extracted with bsbolt CallMethylation, keeping only sites with sequencing depth ≥ 4. Sites common across libraries were identified with bsbolt AggregateMatrix (–min-sample 1.0), and methylation was reported in CGmap format as the fraction of methylated reads at each covered CpG. To generate sample-level profiles, biological replicate CGmap files were consolidated by summing per-position methylated and total read counts. Protein-coding gene coordinates were drawn from GENCODE v44 (comprehensive annotation, hg38). Genomic features were defined as gene bodies, TSS, TTS, and flanking ± 2 kb regions; each feature was tiled into 100-bp bins and CpG sites within each bin were intersected against feature intervals using BEDTools v2.30.0. Per-feature methylation was summarized as a weighted mean (total methylated reads/total reads across all constituent CpG sites), and features with a minimum of 5 total CpG read counts were retained. Metaplots depicting average methylation across gene bodies and flanking regions were generated with ggplot2; all processing and visualization used tidyverse packages in R.

To integrate DNA methylation with transcriptomic data, per-gene methylation values were stratified by differential expression category: genes uniquely upregulated in c42 DND1 + cells relative to d5 hPGCLCs (*n* = 173), genes uniquely upregulated in c42 DND1 − cells relative to d5 hPGCLCs (*n* = 729), and genes shared between both c42 subpopulations relative to d5 hPGCLCs (*n* = 670). Methylation distributions across DEG categories were visualized as violin plots with overlaid boxplots using ggplot2. All data processing and visualization were performed in R using tidyverse packages.

## Results

### The transcriptome of DND1 + in vitro germ cells is enriched with genes associated with RNA regulation

**Fig. 1:**
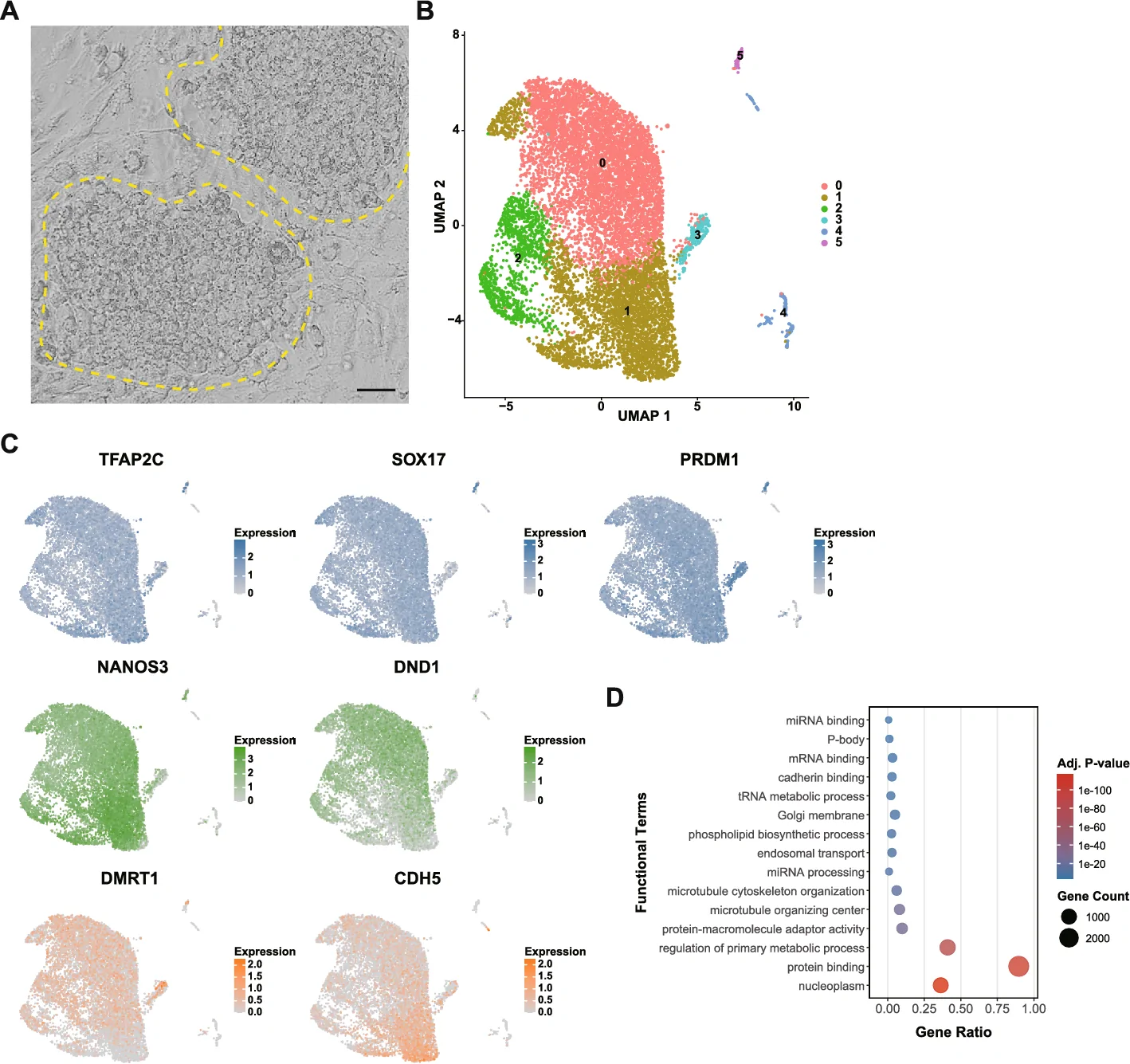
scRNA-seq analysis of hPGCLCs cultured for 21 days in extended culture. **A** Phase-contrast image of hPGCLCs at extended culture day 21 (D4C21). Scale bar = 50 µm. **B** UMAP plot of scRNA-seq from D4C21 showing sub-clusters in different colors. **C** Feature plots for expression of representative genes at D4C21. Top; transcription factors for PGC specification (*TFAP2C*, *SOX17*, *PRDM1*), middle; germ cell-specific RNA binding proteins (*NANOS3*, *DND1*), bottom; pre-gonadal PGC markers (*DMRT1*, *CHD5*). **D** Dot plot presenting functional terms identified for the cluster 0

We first performed single-cell RNA-seq (scRNA-seq) using the 10X Genomics platform to identify gene expression levels in hPGCLCs following 21 days of extended culture (Fig. 1A; D4C21). In these experiments PGCLCs are collected from aggregates after four days of exposure to BMP4 and other cytokines. Two biological replicates were used for scRNA-seq (Table S1). Of the 13,215 cells for which transcriptomes were available, 11,168 were included in the downstream analysis after low-quality cells were removed. To identify clusters of hPGCLCs at day 21 of extended culture, we performed dimensionality reduction and generated a UMAP to display the 11,168 cells. Our initial analysis showed no difference between the two replicates (Fig. S1A), so we combined the cells on a single UMAP revealing 6 sub-clusters (cluster 0 to 5) with clusters 3,4 and 5 representing deviations from the main group composed of clusters 0, 1 and 2 (Fig. 1B). By profiling the expression of known germ cell marker genes, we identified key transcription factors such as *TFAP2C*, *SOX17* and *PRDM1* (Fig. 1C), as well as the pluripotency transcription factors *POU5F1* and *NANOG* (Fig. S1B). These transcription factors together with *NANOS3* were expressed in the three major clusters (0, 1, and 2.). Within the major clusters, *DND1* was enriched in cluster 0 (Fig. 1C). For genes associated with the pre-gonadal state we examined *DMRT1* and *CDH1* [61]. *DMRT1* was detected in clusters 0 and 2, while *CDH5* was specifically found in cluster 1 (Fig. 1C). Gonadal-stage PGC markers, such as *DAZL* and *DDX4*, were not expressed at all (Fig. S1B). We tested for differentially expressed genes by performing a pairwise comparison of each annotated cluster with the others to identify marker genes (Dataset S1). Cluster 0 contained 1,208 marker genes, including the top five genes: *FNDC5*, *DND1* and *GLIPR2* (Dataset S1A). These marker genes were enriched in the Gene Ontology (GO) terms “miRNA binding”, “mRNA binding”, “P-body” and “tRNA metabolic process” (Fig. 1D, Dataset S1B). A total of 1,980 marker genes, including *KRT19*, *ABCA4*, and *SLC38A11*, were found in Cluster 1. Enriched terms included "monoatomic cation transport" and "amide metabolic process" (Fig. S1C, Dataset S1C, D). A total of 1,366 genes were designated as marker genes for cluster 2, including *MKI67*, *CCNA2*, and *CCNA3*. The enriched terms for this cluster were "DNA metabolic process" and "cell cycle process" (Fig. S1C, Dataset S1E, F), indicating a proliferative subpopulation. Overall, this scRNA-seq analysis revealed heterogeneity among hPGCLCs in extended culture with *DND1* expression associated with a PGC state enriched in GO terms for RNA regulation.

### Extended culture leads to progressive enrichment of DND1 + in vitro germ cells

To track *DND1* mRNA translation during in vitro germ cell development, we generated a dual fluorescent reporter hESC line using sequential CRISPR-Cas9-mediated knock-in targeting (Fig. S2A, B). Specifically, we inserted a 2A-eGFP cassette at the *TFAP2C* locus (AG) and a 2A-tdTomato cassette at the *DND1* (DT) locus using the UCLA2 (46,XY karyotype) hESC line. This resulted in a double fluorescence reporter cell line which we call (AGDT). Genotyping PCR confirmed successful targeted integration at both loci across multiple clones, with clone 16 selected for downstream applications (Fig. S2C, red label). Karyotype analysis revealed a normal 46, XY chromosomal complement following genetic modification (Fig. S2D) as well as typical pluripotent stem cell morphology (Fig. S2E). To validate the functionality of the reporter, we differentiated the engineered AGDT hESCs into hPGCLCs through the incipient mesoderm-like cell (iMeLC) intermediate before aggregation in 96-well plates with cytokines to generate aggregates containing hPGCLCs. (Fig. S2F). Using this strategy, we achieved an induction rate of ~ 25% of AG + cells (Fig. S2G). AG + hPGCLCs will now be referred to as hPGCLCs. To confirm that the DND1-2A-TdTomato transgene could be expressed in hPGCLCs we combined the AG + hPGCLCs with mouse embryonic testicular cells to create xenogenic reconstituted testes where hPGCLCs progress to germ cells equivalent to those found in the fetal gonad [49]. At day 2 (d2) of floating culture the hPGCLCs were still negative for DT (Fig. S2H). After transferring the aggregates to an air–liquid interface culture, the aggregates expanded and developed a more complex tubule morphology by day 21 (d2 + 21) (Fig. S2I, top). To evaluate DND1 expression at day 21 we performed flow cytometry analysis which revealed activation of DT + cells within the hPGCLC population. Specifically, by day 21, 7.4% of hPGCLCs now co-expressed DND1-2A-tdTomato (Fig. S2I, bottom). Taken together, using a method that is documented to promote PGC differentiation [49], we show the capacity of specified PGCs to initiate expression of DND1 within the first 21 days of air liquid interface culture. From now on, we will refer to DND1-2A-tdTomato cells as DND1 +.

**Fig. 2:**
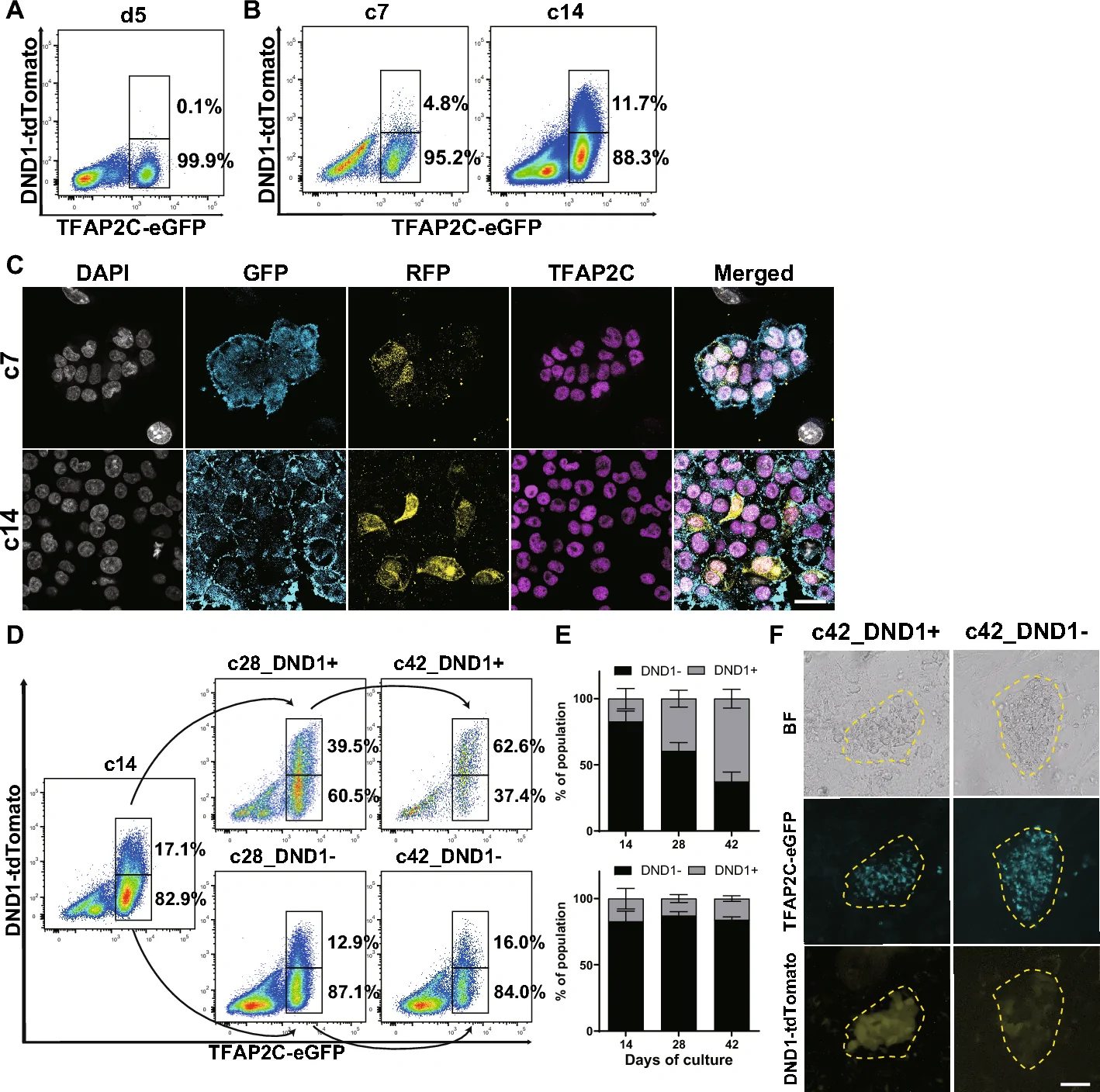
Selecting DND1 + hPGCLCs enriches for DND1 expressing germ cells with time. **A** FACS plots of d5 hPGCLCs (left; d5), and extended culture for 7 days (middle; c7) and 14 days (right; c14). **B** Immunofluorescence (IF) images for TFAP2C (magenta) with TFAP2C-eGFP (GFP; cyan), DND1-tdTomato (RFP; yellow) at c7 and c14. Scale bar, 20 µm and nuclei are stained with DAPI (gray). **D**, **E** FACS plots (**D**) and bar graph (**E**) of a representative extended cultures at c14, and after separation by DND1-tdTomato-positive (DND1 +, top) or negative (DND1-, bottom) at c28 and c42. The average percentage for each population is shown. The arrows indicate enrichment of either DND1 + or DND1- hPGCLCs. Gray and black bars indicate the percentage of DND + and DND1- hPGCLCs respectively at the time points shown. **F** Phase-contrast (BF, left), TFAP2C-eGFP (middle) and DND1-tdTomato (right) epi-fluorescence images of extended culture at c42 with or without enrichment by tdTomato. Yellow dashed lines outline hPGCLC colonies. Scale bar, 50 µm

We next investigated the temporal dynamics of TFAP2C and DND1 expression using the extended culture system. For this approach we isolated hPGCLCs at d5 and plated the cells on mouse STO fibroblasts for up to 42 days of extended culture (c42). At day 5, hPGCLCs had negligible DND1 signal (0.1%) (Fig. 2A). By extended culture day 7 (c7), the hPGCLC population acquired some DND1 (4.8%) (Fig. 2B, left). By c14, this had increased further to 11.7%, (Fig. 2B, right). Microscopic examination of hPGCLCs using epi-fluorescence microscopy revealed AG + hPGCLC colonies at c7 and c14 which increased with size over time (Fig. S2J). Immunofluorescence (IF) analysis confirmed the flow cytometry and epi-fluorescence observations (Fig. 2C). Specifically, at c7, TFAP2C (magenta) was broadly expressed and co-localized with AG (cyan, GFP) in all the hPGCLCs. In contrast, DT (yellow, RFP) was detected in only few cells at c7. By c14, the expression of DT was expressed by individual cells throughout TFAP2C + hPGCLC colonies. We did not observe any colonies that were 100% AGDT double positive at c14. Therefore, DND1 + hPGCLCs are not likely to arise from clonal expansion at c7. Longer-term tracking to c35 resulted in a further increase in the percentage of hPGCLCs acquiring DND1 (22.5%), with modest fluctuations at intermediate time points (c12: 21.1%; c22: 18.5%) (Fig. S2K).

To evaluate the stability of DND1 expression in hPGCLCs, we separated the DND1 + hPGCLCs from the DND- hPGCLCs at c14 using FACS and cultured the two hPGCLC subtypes separately (Fig. 2D–F). At c28 we discovered that most DND1 + hPGCLCs had lost DND1 expression by c28, with only 39.5% of cells remaining double positive. We repeated the FACS separation at c28 and cultured the DND1 + hPGCLCs separately from the DND1- hPGCLCs again for an additional two weeks to c42 (Fig. 2D, E, top). At c42 we discovered more hPGCLCs had retained DND1 expression (62.6%) when a second round of selection was performed. In contrast, when starting with DND1- hPGCLCs at c14 there was a strong bias toward remaining DND1 negative at both c28 and again at c42 (87.1% and 84.0% respectively) (Fig. 2D, E, bottom). Microscopic examination using epi-fluorescence confirmed enrichment of DT (DND1) hPGCLCs throughout the colonies at c42 (Fig. 2F). Collectively, these data indicate that prolonged culture and selection with FACS enriches for hPGCLC of different subtypes as determined using *DND1* translation as a marker. Although DND1 − cells continue to re-emerge from the DND1 + population even after repeated enrichment, this suggests that the DND1 + state is not stably maintained under the current culture condition.

### DND1 + in vitro germ cells exhibit slower growth and increased expression of P27

**Fig. 3:**
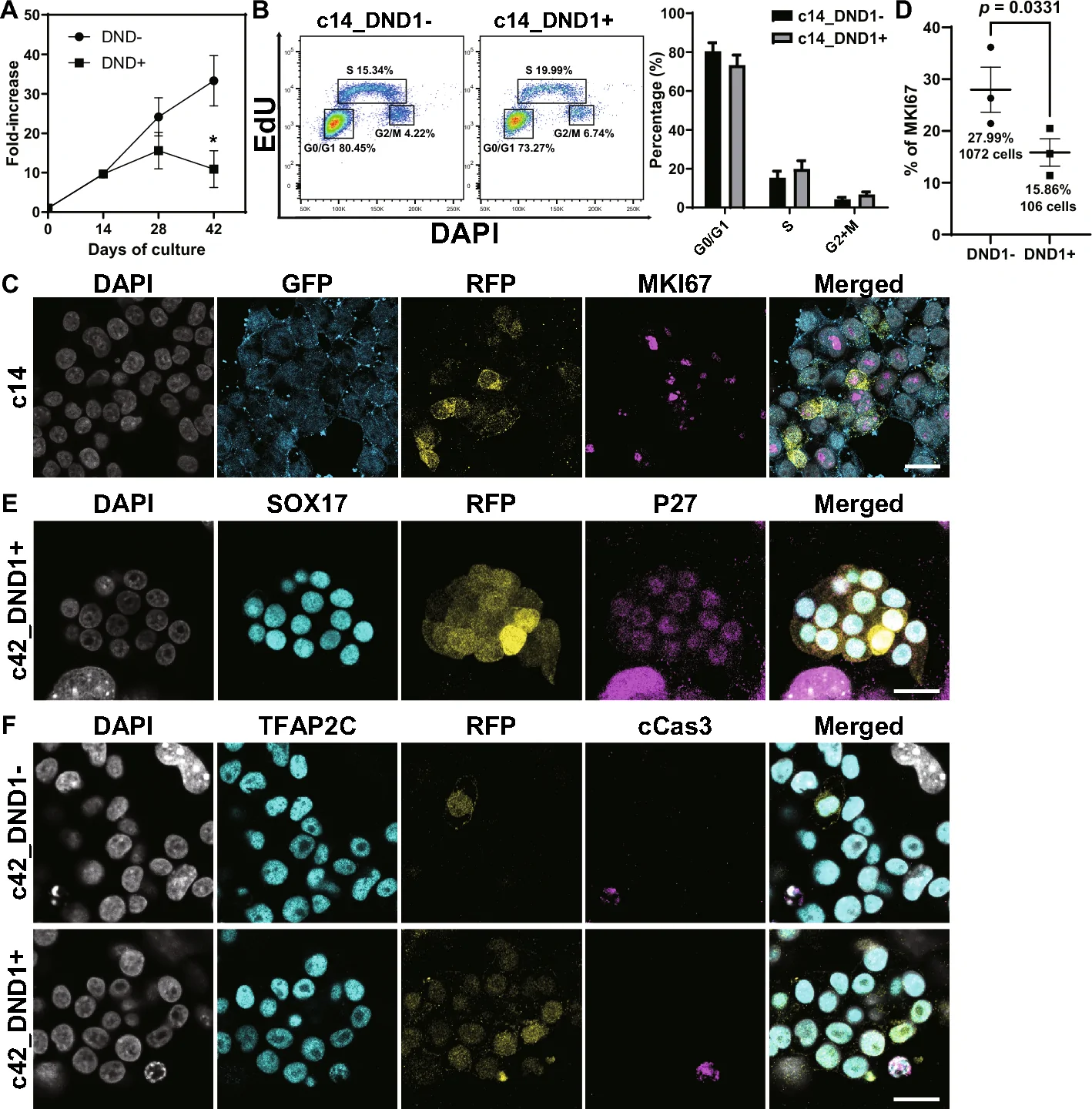
DND1 + hPGCLCs exit the cell cycle. **A** Growth curves of DND1- (circle) or DND1 + (square) hPGCLCs as indicated by log10(fold increases). Error bars, SD. Statistically significant differences between DND1 + and DND1- on c42 were identified with a two-tailed t-test. *p = 0.0472. **B** Cell cycle analysis of DND1- and DND1 + hPGCLCs with EdU and DAPI corporation. (left) EdU and DAPI analysis of DND1 and DND1 + at c14 by FACS. The average percentage of cells in the indicated cell cycle stages are shown. (right) Bar graph of the percentage of each cell cycle stage. Error bars, SD. **C** IF images for MKI67 (magenta) with GFP (cyan) and RFP (yellow) at c14. **D** Quantification of percentage of the MKI67-positive cells in DND1- (GFP + RFP-) or DND1 + at c14. The average percentage and the total number of counted cells are also shown. Statistically significant differences between DND1 + and DND1- hPGCLCs at c42 were identified with Fisher’s exact test, *p* = 0.0331. **E** IF images for P27 (magenta) with SOX17 (cyan) and RFP (yellow) in DND1 + at c42. **F** IF images of cCas3 (magenta) with TFAP2C (cyan) and RFP (yellow) in DND1- and DND1 + hPGCLCs at c42. Scale bars = 20 µm and nuclei are stained with DAPI (gray)

To investigate whether DND1 + hPGCLCs have differential growth rates, we compared the proliferation kinetics of c14 DND1 + and DND1- FACS-isolated hPGCLCs over time. Growth curve analysis revealed that DND1- hPGCLCs exhibited robust expansion, while DND1 + hPGCLCs showed significantly attenuated growth (Fig. 3A). To determine the basis for the differential growth rates, we first performed cell cycle analysis using EdU and DAPI incorporation (Fig. 3B). Starting at c14, flow cytometry revealed modest differences in cell cycle distribution between the two populations (Fig. 3B, left) that were not statistically significant (Fig. 3B, right). At c42, cell cycle dynamics showed a similar trend to c14 (Fig. S3B). Given that cell cycle cannot distinguish between G0 and G1, we performed IF staining for MKI67 which reports cells that are in cycle and this revealed a significant difference between the two subtypes of hPGCLCs (Fig. 3C). Specifically, quantification revealed that 27.9% of DND1- hPGCLCs were MKI67-positive, which was significantly higher (*p* = 0.0331) than the 15.6% of DND1 + hPGCLCs (Fig. 3D). These data indicate that DND1 + hPGCLCs are exiting the cell cycle.

To explore potential mechanisms underlying this, we examined expression of cyclin-dependent kinase (CDK) inhibitors P21 (CDKN1A) and P27 (CDKN1B), key regulators of G0/G1 arrest in germ cells [62, 63]. At c42, DND1 + hPGCLCs showed robust nuclear P27 staining (magenta) that co-localized with SOX17 (Fig. 3E), while P21 was not detected (Fig. S3A, top). The widespread P27 expression in DND1 + hPGCLCs suggests that cell cycle arrest via P27 may contribute to the quiescent phenotype of this PGCLC subtype. In contrast, both P21 and P27 were largely absent in DND1 − hPGCLCs (Fig. S3A, bottom). We also examined whether reduced hPGCLC growth in the DND1 + subtype could be attributed to increased apoptosis. IF for cleaved caspase-3 (cCas3), a marker of apoptotic cells, revealed minimal apoptotic activity in DND1 − and DND1 + hPGCLCs at c42 (Fig. 3F). These findings indicate that differential apoptosis does not account for the differences in population growth dynamics during extended culture. Together, these findings demonstrate that DND1 + hPGCLCs adopt a less proliferative state characterized by reduced MKI67 and upregulation of the cell cycle inhibitor P27.

### DND1 expression status can transcriptionally distinguish in vitro germ cell subtypes

**Fig. 4:**
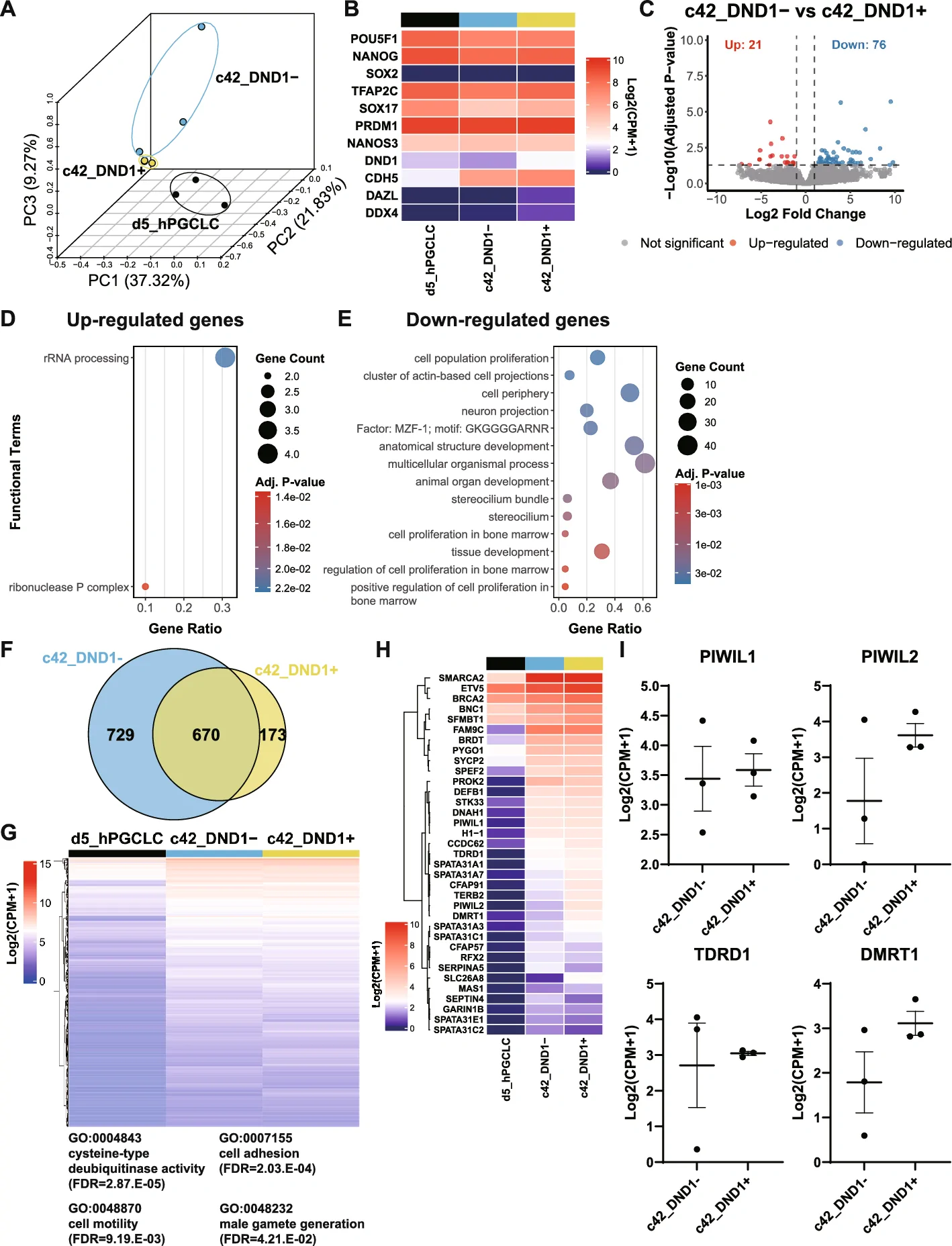
Transcriptomic analysis shows DND1 + and DND1- hPGCLCs are transcriptionally distinct. **A** 3D PCA plot of transcriptomes of d5_hPGCLC (black) DND1- hPGCLCs (cyan) and DND1 + hPGCLCs (yellow) at c42. Color coding indicates cell types. **B** Heatmap of key gene expressions from transcriptomic data. **C** Volcano plot comparing averaged gene expression levels between DND1- and DND1 + hPGCLCs. Red, genes up-regulated; blue, down-regulated in DND1 + compared to DND1- (|logFC|> 1, FDR < 0.05). **D**, **E** Dot plot showing functional terms identified for the up-regulated genes (**D**) and down-regulated genes (**E**) in DND1 + hPGCLCs. **F** Up-regulated genes in DND1- and DND1 + hPGCLCs versus hPGCLC at d5 before extended culture. The number of genes for each category, such as unique to either DND1- or DND1 + hPGCLCs, or shared, are indicated in the Venn diagram. **G** Heatmap of gene expressions from shared up-regulated genes. Representative functional terms are listed below. **H** Heatmap showing gene expressions related to the terms: male gamete generation. **I** Dot plot showing the expression levels of *PIWIL1*, *PIWIL2*, *TDRD1*, and *DMRT1* in DND1- and DND1 + cells. Each dot represents the expression level of one replicate

To characterize the molecular differences between DND1 + and DND1- PGCLCs, we performed bulk RNA-sequencing on serially selected FACS-purified hPGCLC populations at c42. The d5_hPGCLCs were used as a reference. Principal component analysis (PCA) revealed clear transcriptional separation among the three populations (Fig. 4A). The PC1 (37.32%) and PC2 (21.27%) distinguished the extended culture hPGCLCs from d5_hPGCLC, indicating transcriptional changes occur with prolonged culture (Fig. S4A). PC3 (9.27%) separated DND1 + hPGCLCs from DND1 − hPGCLCs (Fig. S4A). Examination of key developmental genes revealed similar and distinct expression patterns when comparing hPGCLC at d5, to the cultured DND1 − and DND1 + hPGCLCs at d42 (Fig. 4B). Pluripotency-associated genes including *POU5F1* and *NANOG*, but not *SOX2*, were robustly expressed in all three populations. Germ cell genes *TFAP2C*, *PRDM1* and *NANOS3* showed sustained expression across all three populations. *SOX17* was slightly decreased in hPGCLCs at c42, although this was not statistically significant. *DND1* was expressed at low levels in hPGCLCs at d5 and DND1- hPGCLCs at c42 with moderate upregulation in DND1 + hPGCLCs. The post-specification gene *CDH5* [61] increased significantly in DND1 + and DND1- hPGCLCs at c42. In contrast, gonadal-stage germ cell markers such as *DAZL* and *DDX4* showed no expression across populations, suggesting that at c42 the germline is still in the pre-gonadal state.

Next, we performed pairwise analysis for differentially expressed genes among three populations. A small number of differentially expressed genes were identified between hPGCLC subtypes at c42 through differential expression analysis, with 21 genes significantly upregulated, and 76 genes downregulated in DND1 + hPGCLCs relative to DND1 − hPGCLCs (|log2FC|> 1, FDR < 0.05) (Fig. 4C, Dataset S2A, B). GO enrichment analysis for upregulated genes in DND1 + cells revealed functional terms related to "rRNA processing" and "ribonuclease P complex," suggesting enhanced ribosomal biogenesis or RNA processing activities (Fig. 4D, Dataset S2C). In contrast, downregulated genes in DND1 + cells were enriched for terms including "cell population proliferation," "cell periphery," "neuron projection," and critically, multiple terms related to cell proliferation and tissue development (Fig. 4E, Dataset S2D). Additional enriched terms included "cluster of actin-based cell projections," "anatomical structure development," and "stereocilium bundle," suggesting that DND1 − hPGCLCs may retain more dynamic cytoskeletal organization associated with active proliferation. Additionally, we compared the DND1 − and DND1 + populations at c42 to the starting hPGCLCs at d5. Compared to d5_hPGCLC, the DND1- cells at c42 showed 1,423 upregulated and 937 downregulated genes, while the DND1 + hPGCLCs at c42 displayed 852 upregulated and 673 downregulated genes (Fig. S4B, Dataset S2E-H, |log2FC|> 1, FDR < 0.05).

Based on pairwise analysis, we categorized the up-regulated genes at c42 in comparison to d5. The Venn diagram showed that 670 genes were commonly upregulated in the extended culture hPGCLC subtypes relative to d5 (Fig. 4F, Dataset S2I). Meanwhile, 729 genes were uniquely upregulated in DND1- hPGCLCS at c42, and only 173 genes were upregulated in the DND1 + hPGCLCs (Fig. 4F and Fig. S4C, Dataset S2I). The 670 shared upregulated genes represent a core transcriptional program acquired during extended culture irregardless of DND1 translation (Fig. 4G). GO analysis of these shared genes identified significant enrichment for terms including "cysteine-type deubiquitinase activity" (GO:0004843, FDR = 2.87E-05), "cell adhesion" (GO:0007155, FDR = 2.03E-04), "cell motility" (GO:0048870, FDR = 9.19E-03), and notably, "male gamete generation" (GO:0048232, FDR = 4.21E-02) (Fig. 4G, Dataset S2J), indicating that extended culture promotes acquisition of molecular features associated with male germ cell development (Fig. 4H). This process involves the coordinated upregulation of multiple components of the PIWI-interacting RNA (piRNA) pathway, including *PIWIL1*, *PIWIL2*, *TDRD1*, and other genes involved in transposon silencing and germline genome defense. Additional male germ cell-associated genes, such as *DMRT1*, *SMARCA2*, *ETV5*, *BRCA2*, *BNC1*, *SPMBTI*, and *FAMC6*, were induced during extended culture. Surprisingly, early meiotic markers, such as *SYCP2*, *SPEF2*, and *PROK2*, exhibited increased expression (Fig. 4H).

We noticed variable expression between biological replicates of hPGCLCs at c42 that were DND- (Fig. S4D). For instance, the mean expression levels of *PIWIL1* and *TDRD1* were comparable between c42_DND1- and c42_DND1 + samples though there was considerable variability between replicates (Fig. 4I). Specifically, *PIWIL2* and *DMRT1* showed more robust induction in DND1 + hPGCLCs with lower and more variable expression in the DND1- hPGCLCs (Fig. 4I). These transcriptomic analyses demonstrate that extended culture drives substantial transcriptional changes in hPGCLCs characterized by the upregulation of piRNA pathway components, male gamete generation programs. DND1 + cells represent a slight advanced developmental state, exhibiting enhanced and stable expression of male germ cell markers of maturing germ cells.

### DND1 + in vitro germ cells have reduced CG methylation levels, particularly at gene bodies

To investigate whether the DND1 + hPGCLCs are associated with DNA demethylation, we performed whole-genome bisulfite sequencing (WGBS) on d5 hPGCLCs and in the serially selected DND1 + and DND1- hPGCLCs at c42. (Fig. S5A). Global CG methylation of d5 hPGCLCs was 73.59% as expected from previous studies [51]. At c42 the hPGCLCs had reduced levels of DNA methylation with DND1 − hPGCLCs exhibiting 64.81% CG methylation and DND1 + hPGCLCs exhibiting even lower CG methylation at 61.04%. Indicating that the global DNA methylation difference between the two subpopulations was modest. Although lower than d5, the CG methylation levels of c42 hPGCLCS were still considerably higher than hPGCs at W5.5 (21.15%). Examination of overall CG methylation distributions across the genome revealed the extent of demethylation in c42 hPGCLCs (Fig. S5B, Dataset S3A). Violin plots showed that d5_hPGCLC displayed a distribution with the highest observed methylation (60.72%), while c42_DND1 − exhibited intermediate methylation (55.80%), and c42_DND1 + showed the lowest methylation (47.47%) among the in vitro populations. Thus, a progressive reduction in methylation levels was shown from d5 through c42, with the DND1 + hPGCLCs at c42 exhibiting the most advanced state of DNA demethylation. Next, we analyzed CG methylation patterns across all annotated genes and their flanking regions (Fig. S5C, Dataset S3B). Here, we discovered that DND1 + hPGCLCs are the most demethylated subtype particularly at gene bodies, even when compared to DND- hPGCLCs. Furthermore, we stratified per-gene gene body methylation values by differential expression category (Fig. 4F). Gene body methylation was consistently lowest in c42 DND1 + cells across all DEG categories examined, while c42 DND1 − cells exhibited higher gene body methylation than d5 hPGCLCs at these same loci (Fig. S5D, Dataset S3C). These findings support the interpretation that DND1 + hPGCLCs represent the most epigenetically demethylated state both globally and particularly at the gene bodies of differentially upregulated genes in DND1 + cells.

### BMP2 stabilizes DND1 translational in extended culture

**Fig. 5:**
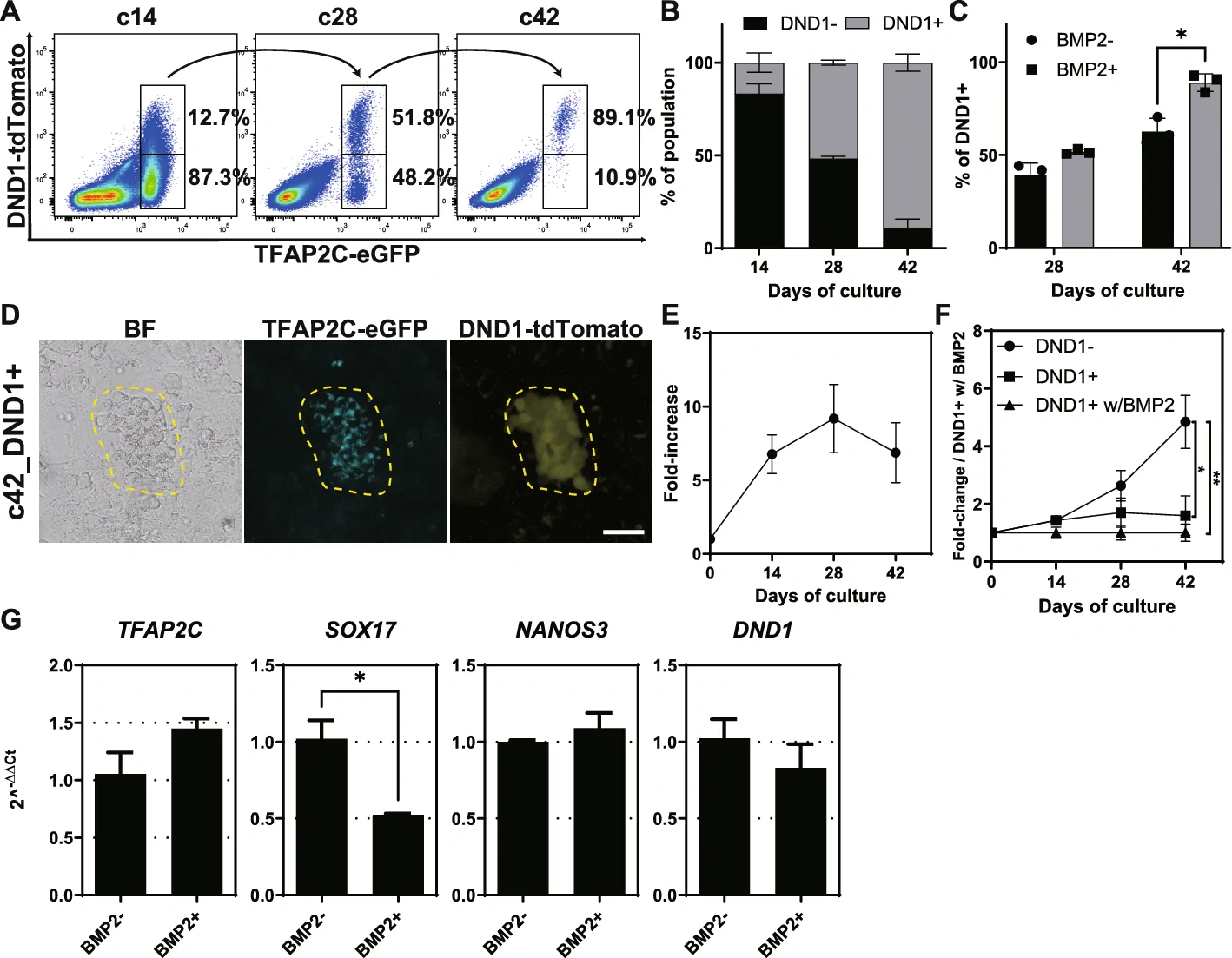
BMP2 stabilizes DND1 translation between c28-c42. **A**, **B** FACS plots (**A**) and bar graph (**B**) of DND1 + hPGCLC cultured with BMP2 from c14-c42. The average percentage for each population is shown. Gray and black bars indicate the percentage of DND + and DND1- hPGCLCs during the culture, respectively. Error bars, SD. **C** Bar graph comparing the percentage of DND1 + hPGCLCs at c28 and c42 with or without BMP2 (BMP2- or BMP2 +). Each dot represents one replicate. Error bars, SEM. Statistically significant differences between BMP2- and BMP2 + on c42 were identified with a two-tailed t-test. *p = 0.0003. **D** Phase-contrast and epi-fluorescence images of DND1 + hPGCLCs with BMP2 at c42. Cyan, TFAP2C-eGFP; yellow, DND1-tdTomato. Yellow dotted lines highlights colonies of hPGCLCs. Scale bar = 50 µm. **E** Growth curve of DND1 + hPGCLCs cultured with BMP2 as indicated by log10(fold increases). **F** Relative fold-change of growth curve for DND1- (circle) and DND1 + (square) hPGCLCs cultured without BMB2 versus DND1 + hPGCLCs with BMP2 (triangle). Error bars, SEM. Statistically significant differences were identified with One-way ANOVA. *p = 0.0468, **p = 0.0100. **G** Relative gene expression analysis of germ cell-expressed genes at c42 in DND1 + hPGCLCs cultured with and without BMP2. qPCR was conducted in triplicate and normalized to expression of the average of *ACTB* and *GAPDH*. Statistically significant differences were identified with a two-tailed t-test. *p = 0.0150

Given that BMP signaling promotes epigenetic reprogramming and further differentiation of hPGCLCs [53], we investigated the effect of BMP2 signaling on DND1 expression in our system. We first established hPGCLC cultures from d5 hPGCLCs with BMP2 included from the beginning to culture. Analysis at c14 yielded lower DT expression (6.0%) compared to previous culture without BMP2 (Fig. S6A, B). Therefore, we added BMP2 to the media from c14 with analysis at c28 and c42 (Fig. 5A). Strikingly, BMP2 treatment from c14-c28 resulted in a higher percentage of DND1 + hPGCLCs by c42 with 89.1% of the hPGCLC population now expressing DND1 which was further increased at c42 (Fig. 5A, B). Comparing percent DND1 + hPGCLCs in the presence and absence of BMP2 reveals a significant increase in the proportion of DND1 + hPGCLCs specifically at c42 (Fig. 5C). Epifluorescence microscopic examination of BMP2-treated DND1 + cultures at c42 revealed robust co-expression of AG (cyan) and DT (yellow) in hPGCLC colonies (Fig. 5D). This indicates that BMP2 signaling is critical for maintaining DND1 + expression in hPGCLCs, specifically after c28.

To investigate proliferation of the DND1 + cells, we performed growth curve analysis (Fig. 5E). BMP2-treated DND1 + cells achieved an eightfold increase by c28. However, growth of DND1 + cells between c28-c42 was still attenuated (Fig. 5E). When comparing relative growth rates among DND + hPGCLCs with and without BMP2, our results show that BMP2 has no statistically significant effect on the growth rate of DND1 + hPGCLCs (Fig. 5F). These data demonstrates that the increased proportion of DND1 + hPGCLCs in BMP2-treated cultures reflects maintenance of DND1 expression and not an increase in proliferation leading to a greater proportion of DND1 + cells. Given that BMP2 lead to an increase in the % of DND1 + hPGCLCs at c42, we next evaluated whether this was associated with an increased in *DND1* RNA expression (Fig. 5G). Using semi-quantitative RT-PCR at c42 our data shows that *TFAP2C* and *NANOS3* levels are similar between groups, However, *SOX17* was significantly downregulated (*p* = 0.0150), while *DAZL* and *DDX4* were at the limit of detection regardless of BMP2 at c42 (Fig. S6C). Analysis of *DND1* shows no statistical difference between BMP2 treated or control groups, suggesting that BMP2 likely operates by preventing the reversion of DND1 + hPGCLCs to the DND1-state rather than increasing the expression of *DND1* RNA levels. This result indicates that BMP2 supplementation can significantly improve the efficiency of translation of target RNAs, as well as generating matured germ cell populations in vitro [53].

## Discussion

As the progenitors of eggs and sperm, PGCs represent the earliest cell type committed to the germline, the singular lineage entrusted with conveying genetic and epigenetic information between generations. Proper genetic and epigenetic material transfer through germ cells is critical for offspring health. To ensure faithful transmission of genetic material, germ cells undergo dynamic and carefully orchestrated developmental transitions during embryogenesis. A distinctive feature of germ cell maturation is the coordinated cell cycle, which may provide a temporal window for epigenetic reprogramming and serves as a critical checkpoint for maintaining germline genome integrity and genetic stability [3, 19, 20, 32–34]. Unlike mice, in which the developmental stage and cell cycle are uniformly regulated in fetal germ cells [20, 62], human fetal germ cells exhibit heterogenous character [35, 49]. However, it was not clear whether this heterogeneity endowed human fetal germ cells with distinct biological properties until now using hPGCLCs as a model. Direct in vivo validation of PGCs at comparable human fetal stages remains challenging given the inaccessibility of this developmental window.

Using the in vitro extended culture system of hPGCLCs, our single-cell RNA sequencing analysis revealed distinct sub-populations of in vitro germ cells with markedly different proliferative and molecular characteristics. Cluster 2 (DND1-low) represents a highly proliferative state, whereas cluster 0 (DND1-high) exhibited pronounced enrichment for GO terms related to miRNA processing, P-bodies, tRNA metabolism, and Golgi membrane. This molecular signature suggests that DND1 + sub-populations have transitioned from active proliferation to a post-transcriptionally regulated state characterized by enhanced post-transcriptional and translational control mechanisms. The enrichment of P-body-associated genes in DND1 + hPGCLCs is particularly notable, as P-bodies serve as phase-separated sites for mRNA storage, decay, and translational repression [64], suggesting that DND1 + cells could actively remodel their repertoire of RNAs and consequently their proteome through post-transcriptional mechanisms. The concurrent enrichment of Golgi and tRNA-related genes further implies that these cells are preparing for specialized protein synthesis and secretory functions characteristic of maturing germ cells. Notably, heterogeneous DND1 expression has also been described in late fetal male germ cells in mice [36], supporting the biological plausibility of heterogeneous DND1 translation in human fetal germ cells. However, it remains to be determined whether equivalent states exist in the human PGCs in vivo. Consistent with their transcriptionally distinct state, DND1 + hPGCLCs also exhibit more advanced DNA demethylation than DND1 − hPGCLCs, particularly at gene bodies of differentially upregulated genes. Methylation levels at c42 remain considerably higher than those of Wk5.5 PGCs in vivo, indicating that the PGCLCs these cultures at c42 occupy an intermediate epigenetic state.

RNA expression in human germ cells has been extensively studied due to the compatibility of transcriptomics technologies with the study of rare cell populations such as PGCs. In contrast, the post-transcriptional regulatory landscape in human germ cells in vitro and in vivo is less well known. Post transcriptional regulation is orchestrated primarily by RNA-binding proteins (RBPs) and microRNAs, which play indispensable roles in controlling mRNA stability, subcellular localization, and translational efficiency. We show that DND1 is an initial marker of an altered RNA regulatory state. This is an important observation due to DND1’s role as a central coordinator with evolutionarily conserved functions essential for PGC survival and tumor suppression in mice and zebrafish [42, 43]. The inverse correlation between DND1 expression and proliferative capacity observed in our study suggests that DND1 may function as a developmental checkpoint to suppress proliferation while simultaneously promoting molecular maturation. This interpretation is consistent with recent findings that DND1, in collaboration with NANOS3, regulates the translation of key developmental regulators such as SOX4 within P-bodies, thereby restricting premature or inappropriate cell fate transitions [65]. Together, these observations could imply that the heterogeneity observed in hPGCLC populations reflects distinct developmental states along a continuum from proliferative progenitors to post-transcriptionally primed DND1 + cells preparing for subsequent differentiation. Whether DND1 serves as a critical orchestrator of this developmental transition or a useful marker remains to be determined.

Our study of in vitro germ cell development revealed that DND1 translation is associated with a distinct biological state. Examples of distinct biological states have been observed before in the male germline using transgenic mice for Id4-GFP. In these mice Id4-GFP + male spermatogonial cells have a robust regenerative capacity when transplanted into recipient testes whereas Id4-GFP- spermatogonia from the same animal do not, even though the transcriptomic differences were subtle [66]. This apparent discordance between transcriptome and phenotype underscores the critical importance of post-transcriptional regulatory mechanisms or other physiological controls in determining cellular identity and function. The enrichment of translation-related functional terms specifically in DND1 + cells suggests active remodeling of the translational machinery rather than merely altered transcript abundance. Consistent with this interpretation, DND1 has been demonstrated to physically interact with both translational machinery components and chromatin modifiers [36], positioning it as a potential integrator that coordinates post-transcriptional control with chromatin state transitions during germ cell maturation.

Notably, DND1 + cells displayed significantly elevated expression of ribosomal RNA processing-related genes, including *RPS13*, *RPP30*, and *RPL27*, suggesting differential utilization of ribosomal protein composition between DND1 + and DND1- PGCLC populations. Recent evidence has established that ribosome heterogeneity, which arises from variations in ribosomal protein stoichiometry, paralog composition, and post-translational modifications, endows ribosomes with selective translational capacity for specific mRNA subpools. The selective upregulation of specific ribosomal protein genes in DND1 + hPGCLCs raises the intriguing possibility that these cells assemble specialized ribosomes tailored for the translation of germ cell differentiation programs. Indeed, DND1 + cells exhibited more stable and elevated expression of critical germ cell differentiation-related genes, such as *PIWIL1*, *PIWIL2*, *TDRD1*, and *DMRT1*, all of which encode proteins essential for germline identity and function. However, direct interactions still need to be validated. Despite modest changes at the transcriptional level between DND1- and DND1 +, this stable expression pattern may suggest that post-transcriptional and translational control mechanisms could play an important role in maintaining the germ cell gene expression program.

DND1 functions as a central post-transcriptional regulator in mouse PGCs, orchestrating gene expression through direct mRNA binding and recruitment of regulatory complexes. Our observation that DND1 + hPGCLCs exhibit nuclear expression of P27 (CDKN1B), coupled with reduced proliferation and exit from cell cycle, aligns with recent mechanistic insights demonstrating that *P27* is a direct binding target of human DND1 [65]. Studies in mouse germ cells have demonstrated that Dnd1 directly binds *P27* and *P21* (*Cdk1a*) RNA, and that loss of *Dnd1* is associated with a significant decreases in both P27 and P21 proteins across all strain tested, suggesting that DND1 regulates cell cycle arrest in fetal male germ cells through translational regulation [63]. The preservation and upregulation of P27 protein levels in our DND1 + human cells suggests that DND1 selectively promotes translation or stability of *P27* mRNA in hPGCLCs. The resulting P27-mediated cell cycle exit provides a molecular explanation for the reduced proliferative capacity observed in DND1 + cells relative to DND1-. Whether additional forms of programmed cell death, including autophagy or necroptosis, contribute to the attenuated growth of DND1 + hPGCLCs was not assessed in this study. Given established roles for autophagy in germ cell homeostasis [67], these pathways represent important avenues for future investigation.

Recent advances demonstrate that sustained BMP2 supplementation in long-term hPGCLC culture promotes epigenetic reprogramming and further differentiation [53]. In our extended culture system, BMP2 supplementation increased DND1 translation as reported using 2A-TdTomato, without corresponding changes in *DND1* mRNA abundance. The metastability of the DND1 + state, as observed following FACS enrichment where cells progressively revert to DND1 − in the absence of exogenous signaling, is consistent with this model. Our data supports the hypothesis that sustained BMP2 activity may be necessary to maintain the translational competence required for DND1 protein production. BMP2 binding to its receptors (BMPR1A/1B and BMPR2) activates both canonical SMAD1/5/8-dependent and non-canonical SMAD-independent pathways [68, 69]. Downstream of BMP2 signaling, mTOR activity can selectively enhance the translation of specific transcripts through 4E-BP1-mediated regulation of eIF4E availability, particularly mRNAs bearing 5' terminal oligopyrimidine (TOP) motifs, without necessarily driving a global increase in protein synthesis [70]. Therefore, future experiments could test whether BMP2’s effect on increasing translation capacity in the human germline extends beyond DND1.

## Conclusions

In summary, we investigated the molecular and functional heterogeneity of hPGCLCs in extended culture, with a focus on *DND1* RNA translation dynamics using 2A-TdTomato. We demonstrated that DND1 + cells represent a distinct developmental state characterized by reduced proliferative capacity, cell cycle exit and upregulation of P27. Additionally, we observed BMP2 supplementation stabilized DND1 translation without enhancing RNA levels or proliferation. These findings provide valuable insights on the importance of post-translational modifiers of germ cell biology. We predict that the next frontier in advancing our understanding of human germline development should involve detailed characterize of the human germline proteome.

## Supplementary Information

Additional file 1. Figure S1. scRNA-seq of hPGCLC extended culture. UMAP plot of scRNA-seq from two replicates of D4C21 Feature plots for representative gene expression from D4C21. Top; transcription factors for pluripotency, bottom; post-migratory PGC markers. Dot plot presenting functional terms identified for the cluster 1and 2. Figure S2. Generation and validation of TFAP2C-eGFP and DND1-tdTomato dual reporter hESC line. Schematic representation of CRISPR-Cas9 targeting strategy for TFAP2Cand DND1loci. The constructs for knocking-in 2A-eGFP into the TFAP2C or 2A-tdTomato into the DND1 loci are also shown. Black boxes indicate the exons. Arrows indicate PCR screening primers. Genotyping PCR analysis of targeted clones for TFAP2C, DND1, and random integration of the targeting vector. Rec., recombined by Cre; non, non-targeted. Yellow arrow, 4 kb; yellow asterisk, 1.5 kb. Clone 16was selected for the AGDT hESC line Representative karyotype for AGDT showing normal 46, XY chromosomal complement. Phase-contrast image of AGDT hESC line. Phase-contrast image of AGDT-derived iMeLCs. Phase-contrastand epi-fluorescenceimages of hPGCLCs derived from AGDT hESC for TFAP2C-eGFP Phase-contrast imagesand flow cytometry analysisof xrTestes aggregates at d2 and ALI culture at d2 + 21 . Phase-contrastand TFAP2C-eGFP epi-fluorescenceimages of extended culture at c7 and c14. Yellow dashed lines outline TFAP2C-eGFP + hPGCLC colonies. Flow cytometry analysis of TFAP2C-eGFP and DND1-tdTomato expression during extended culture at c12, c22, and c35 without enrichment by tdTomato. Scale bars in E-I, 200 μm. Scale bar in J, 50 μm. Figure S3. Immunostaining and cell cycle analysis in extended culture at c42. IF images for P21with TFAP2Cand RFPin DND1 + at c42. IF images for P21and P27with TFAP2C-eGFPin DND1- hPGCLCs at c42. Scale bars = 20 µm and nuclei are stained with DAPI. Cell cycle analysis of DND1- and DND1 + hPGCLCs by EdU and DAPI corporation at c42.EdU and DAPI analysis of DND1 and DND1 + hPGCLCs by FACS. The average percentage of cells in the indicated cell cycle stages are shown.Bar graph of the percentage of each cell cycle stage. Error bars, SD. Figure S4. Comprehensive transcriptomic comparisons during extended culture. 2D PCA plots showing pairwise comparisons of transcriptomes. Left: PC1 vs PC2; Middle: PC1 vs PC3; Right: PC2 vs PC3. Black circles, d5_hPGCLC; cyan circles, c42_DND1 −; yellow circles, c42_DND1 +. Volcano plots comparing gene expression between d5_hPGCLC and extended culture populations. Left: d5_hPGCLC vs c42_DND1 −; Right: d5_hPGCLC vs c42_DND1 +. Red dots indicate up-regulated genes; blue dots indicate down-regulated genes. Numbers of differentially expressed genes are shown. Heatmap showing expression patterns of all differentially expressed genes unique to c42_DND1 − and c42_DND1 + hPGCLCs, compared to hPGCLC at d5. Hierarchical clustering reveals distinct transcriptional programs. Heatmap of genes associated with male gamete generation across biological replicates of d5 and c42 DND1 −, and DND1 + hPGCLCs. Gene names are listed on the right. Color scale represents Log2. Figure S5. DNA methylation status in extended culture. Dot plot showing global CG methylation levels in hPGCLCs at d5, DND1-, DND1 + hPGCLCs at c42 and female hPGCs at week 5.5. Statistically significant differences were identified with One-way ANOVA. *p = 0.0057 between d5_hPGCLC and c42_DND1 +, *p < 0.0001 compared to hPGC Wk5.5 F. The DNA methylome data of hPGC is from SRP057098. The whole-genome bisulfite sequencing analysis covered 22.7 K CpG sites in d5_hPGCLC, 10.6 K CpG sites in c42_DND1 − , and 5.7 K CpG sites in c42_DND1 + . Violin plots for the distribution of overall CG methylation level. Average CG methylation levels over protein coding genes and 2 kb flanking regions TSS, transcription start site; TTS, transcription termination site. CpG methylation at gene bodies of differentially expressed gene sets. Figure S6. BMP2 supplement on hPGCLC extended culture. A FACS plot and a bar graph of hPGCLC cultured with BMP2 from c0-c14. The average percentage for each population is shown. Gray and black bars indicate the percentage of DND + and DND1- hPGCLCs, respectively. Error bars, SD. Gene expression analysis of *DAZL* and *DDX4* at c42 in DND1 + cells cultured with and without BMP2. qPCR was conducted in triplicate and normalized to expression of the average of *ACTB* and *GAPDH*. Error bars, SEM. Statistically significant differences were identified with a two-tailed t-test. ns, non-significance.

Additional file 2. Table S1. Quality Metrics of 10 × data sets

Additional file 3. Table S2. List of antibodies

Additional file 4. Table S3. List of TaqMan primers

Additional file 5. Dataset S1. Differentially expressed genes and functional terms in each cluster by pair-wise comparison in single-cell RNA-seq

Additional file 6. Dataset S2. Differentially expressed genes and functional terms by pair-wise comparison and Venn diagram categorization in bulk RNA-seq

Additional file 7. Dataset S3. Statistics of whole-genome bisulfite sequencing

Additional file 8. Source Data 1. Full uncropped Gels for Figure S2C

## Data Availability

scRNA-seq, bulk RNA-seq and WGBS data in this study have been deposited at Gene Expression Omnibus (GEO) with the following accession numbers GEO: GSE313385, GSE313384 and GSE313386, and are publicly available as of the day of the publication.

## Abbreviations

PGC: Primordial germ cell
wpc: Week post-conception
DND1: Dead end 1
RBP: RNA binding protein
IVG: In vitro gametogenesis
hPGCLCs: Human primordial germ cell-like cells
hESC: Human embryonic stem cell
AG: TFAP2C-p2A-eGFP
DT: DND1-p2A-tdTomato
iMeLCs: Incipient mesoderm-like cells
MMC: Mitomycin C
scRNA-seq: Single cell RNA-seq
GEM: Gel beads-in-emulsion
PCA: Principal component analysis
UMAP: Uniform manifold approximation and projection
GO: Gene Ontology
KEGG: Kyoto Encyclopedia of Genes and Genomes
NDS: Normal donkey serum
DEG: Differentially expressed genes
WGBS: Whole-genome bisulfite sequencing
gDNA: Genomic DNA
cCas3: Cleaved caspase-3
piRNA: PIWI-interacting RNA
