# Supplementary material for: Human primordial germ cell heterogeneity in vitro is associated with distinctive biological states
Source: Cell Mol Biol Lett. 2026 Jun 24;31:142. doi: 10.1186/s11658-026-00986-w (PMC13560305; doi:10.1186/s11658-026-00986-w)
Supplement: Supplementary file 1 [file 11658_2026_986_MOESM1_ESM.pdf]

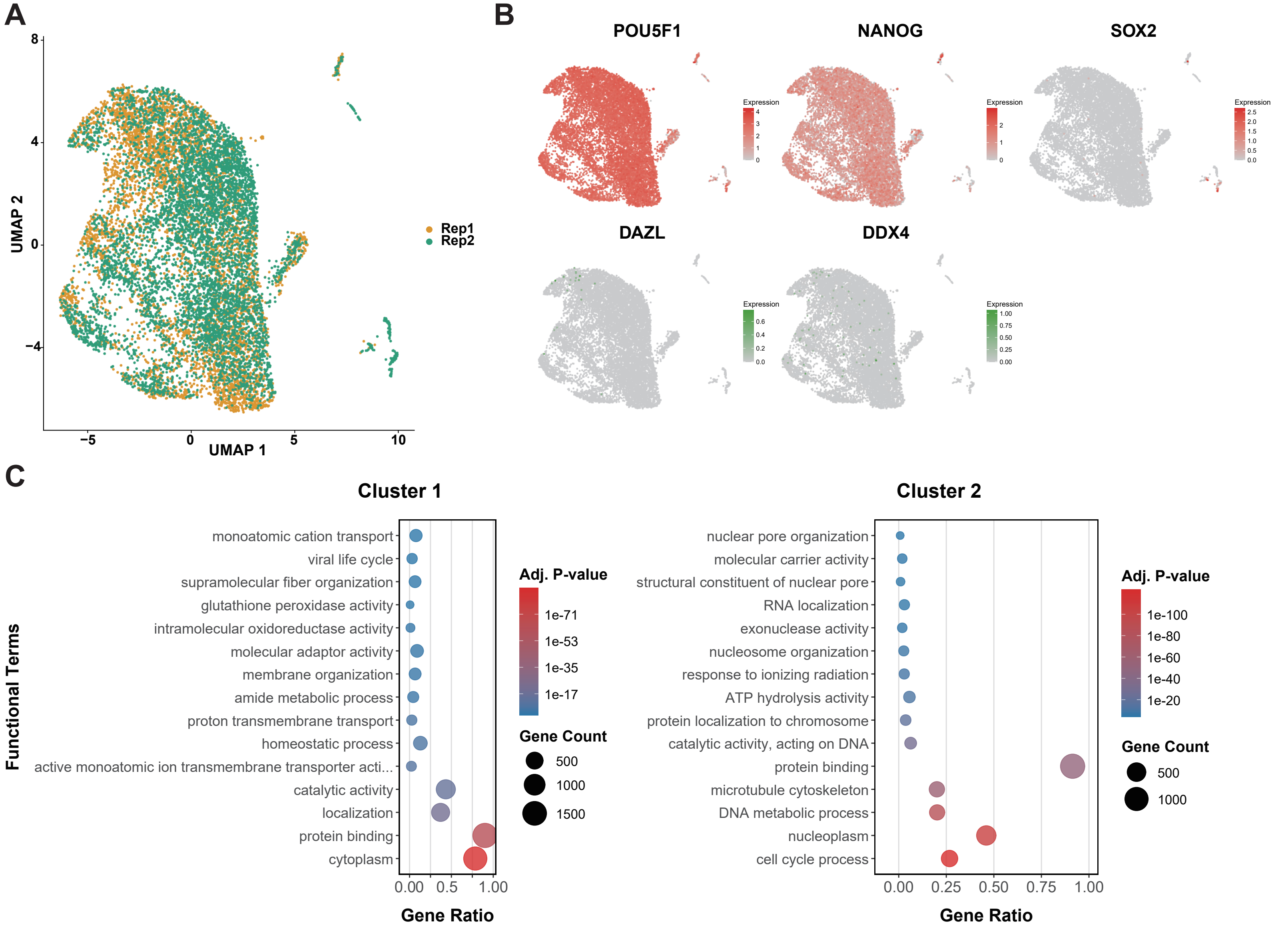

A
B
C
UMAP 1
UMAP 2
Rep1
Rep2
POU5F1
NANOG
SOX2
DAZL
DDX4
Expression
Cluster 1
Cluster 2
Functional Terms
monoatomic cation transport
viral life cycle
supramolecular fiber organization
glutathione peroxidase activity
intramolecular oxidoreductase activity
molecular adaptor activity
membrane organization
amide metabolic process
proton transmembrane transport
homeostatic process
active monoatomic ion transmembrane transporter acti...
catalytic activity
localization
protein binding
cytoplasm
Gene Ratio
Adj. P-value
1e-71
1e-53
1e-35
1e-17
Gene Count
500
1000
1500
nuclear pore organization
molecular carrier activity
structural constituent of nuclear pore
RNA localization
exonuclease activity
nucleosome organization
response to ionizing radiation
ATP hydrolysis activity
protein localization to chromosome
catalytic activity, acting on DNA
protein binding
microtubule cytoskeleton
DNA metabolic process
nucleoplasm
cell cycle process
Adj. P-value
1e-100
1e-80
1e-60
1e-40
1e-20
Gene Count
500
1000

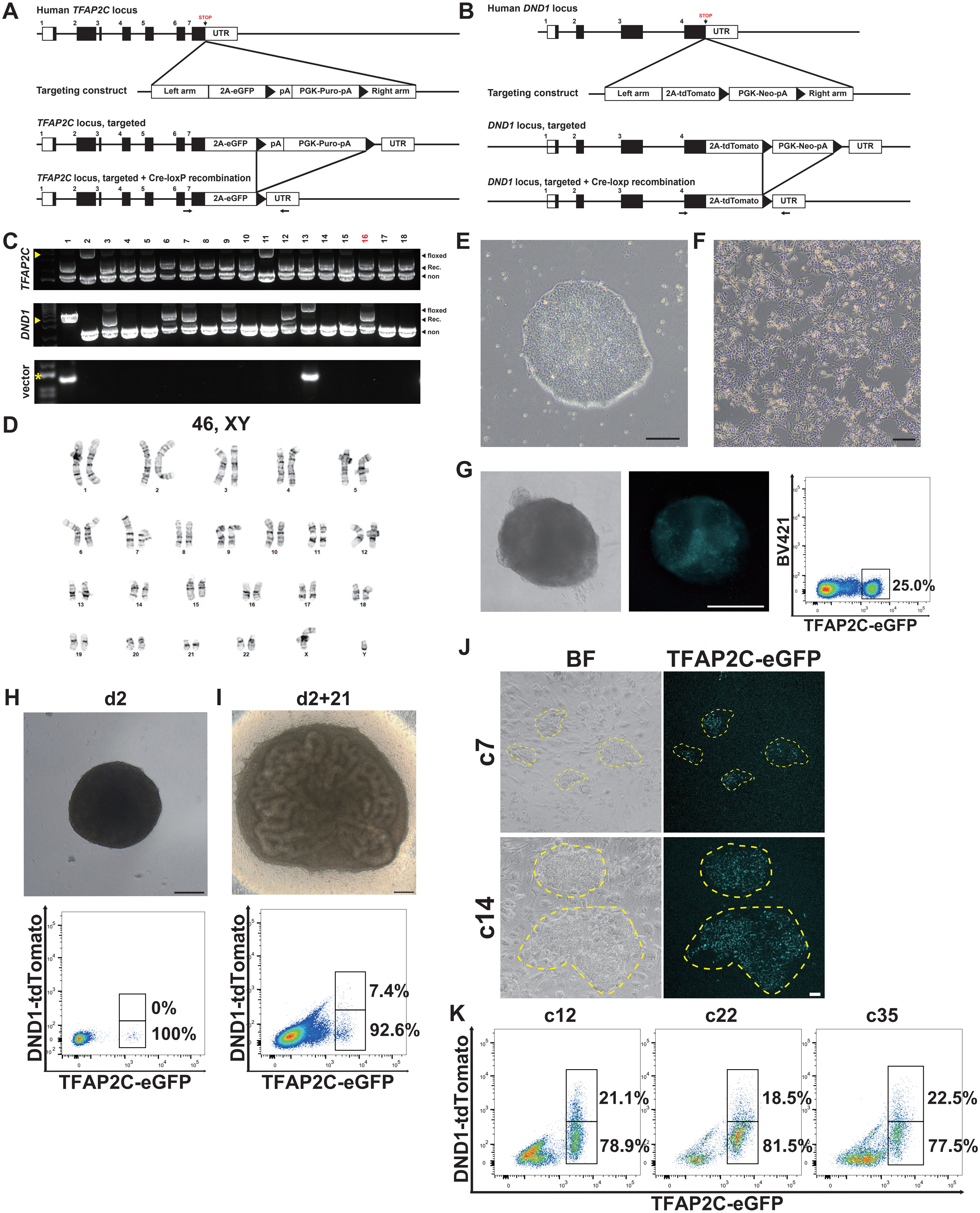
A
Human TFAP2C locus
STOP
UTR
Targeting construct
Left arm
2A-eGFP
pA
PGK-Puro-pA
Right arm
TFAP2C locus, targeted
TFAP2C locus, targeted + Cre-loxP recombination
B
Human DND1 locus
2A-tdTomato
PGK-Neo-pA
DND1 locus, targeted
DND1 locus, targeted + Cre-loxp recombination
C
TFAP2C
DND1
vector
floxed
Rec.
non
D
46, XY
E
F
G
BV421
TFAP2C-eGFP
25.0%
H
d2
DND1-tdTomato
0%
100%
I
d2+21
7.4%
92.6%
J
BF
TFAP2C-eGFP
c7
c14
K
c12
c22
c35
21.1%
78.9%
18.5%
81.5%
22.5%
77.5%

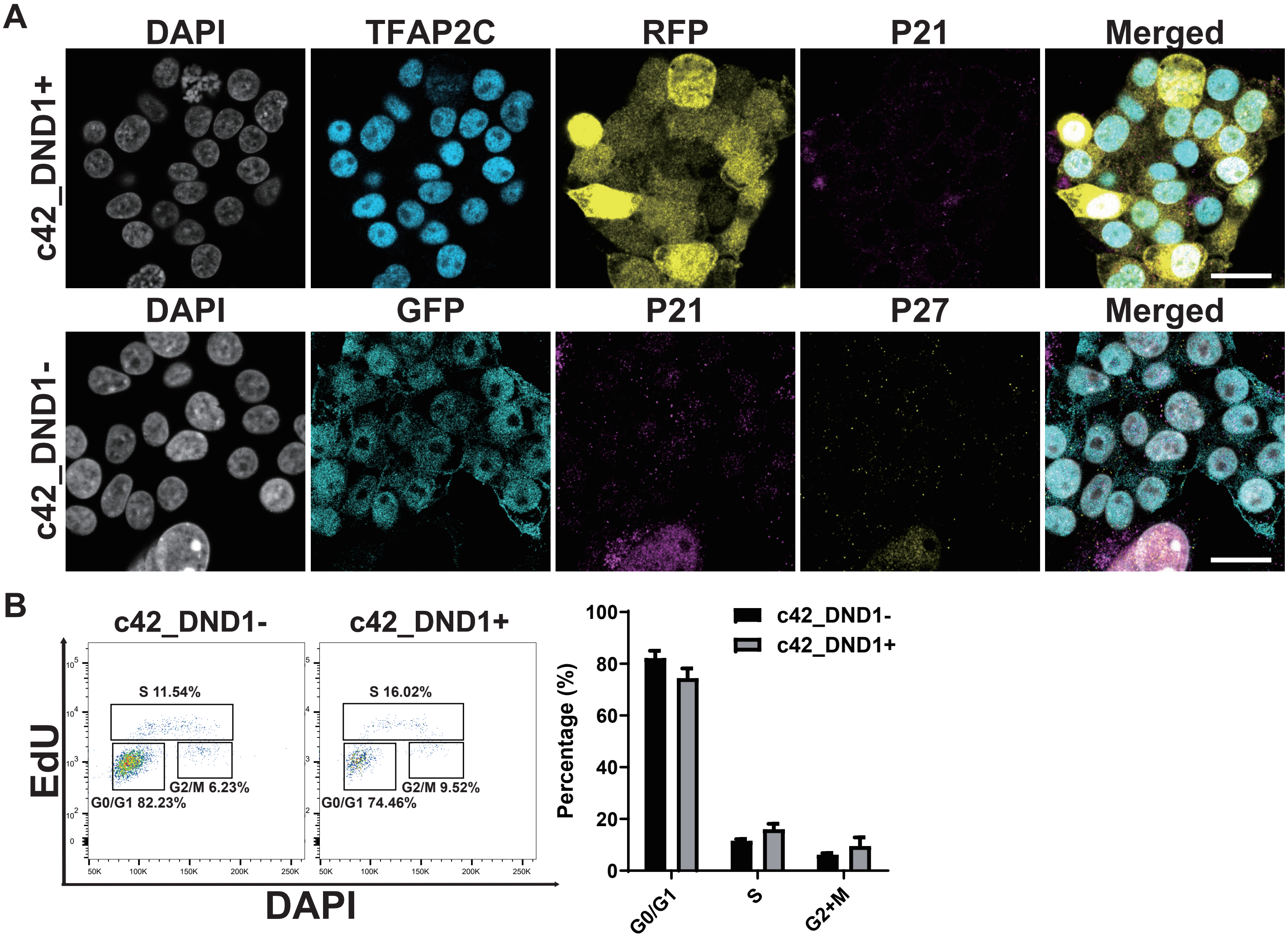
A
DAPI
TFAP2C
RFP
P21
Merged
c42_DND1+
DAPI
GFP
P21
P27
Merged
c42_DND1-
B
c42_DND1-
c42_DND1+
S 11.54%
G2/M 6.23%
G0/G1 82.23%
S 16.02%
G2/M 9.52%
G0/G1 74.46%
EdU
DAPI
50K 100K 150K 200K 250K
Percentage (%)
c42_DND1-
c42_DND1+
G0/G1
S
G2+M
0
20
40
60
80
100

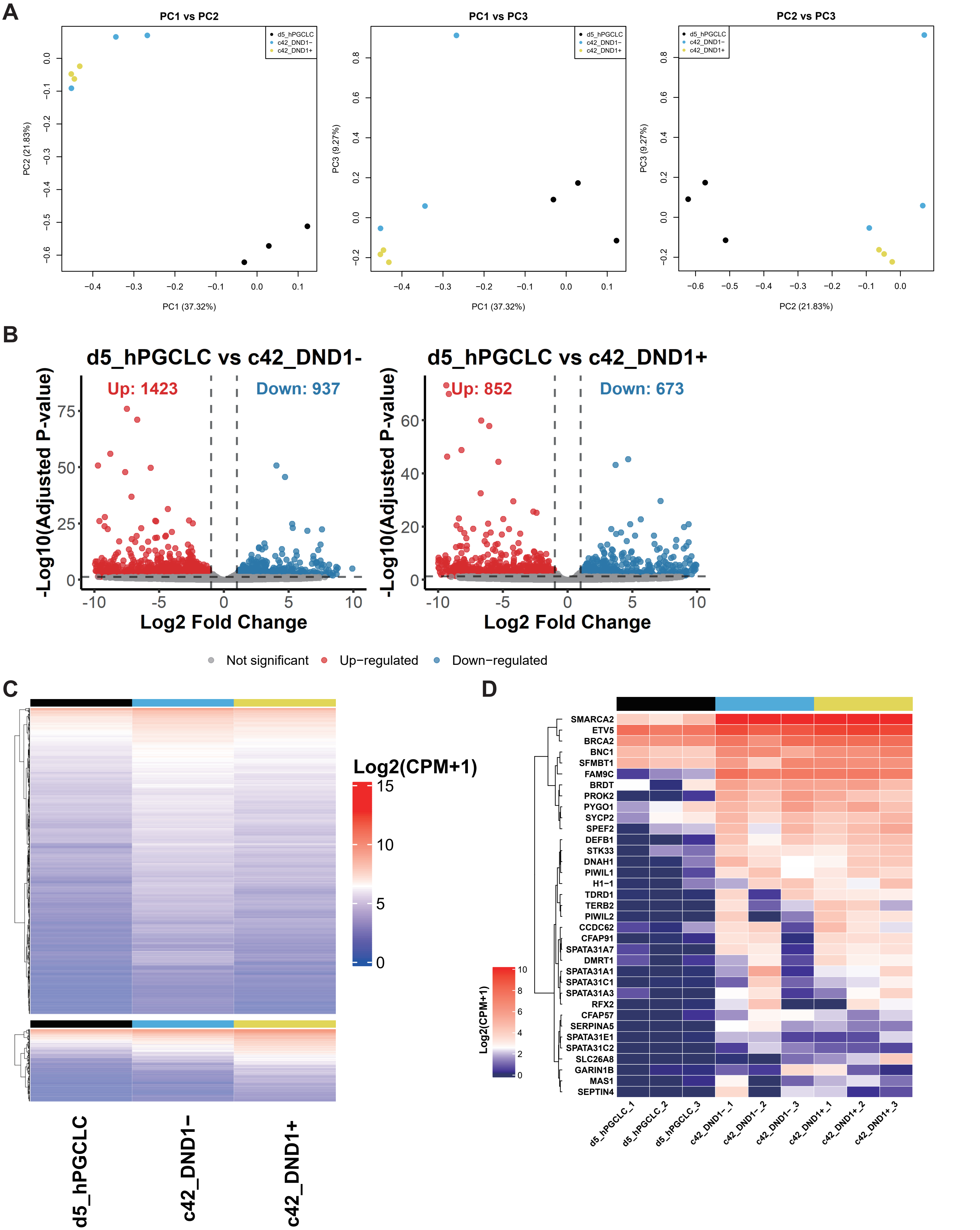

A
PC1 vs PC2
PC1 vs PC3
PC2 vs PC3
d5_hPGCLC
c42_DND1−
c42_DND1+
PC1 (37.32%)
PC2 (21.83%)
PC3 (9.27%)
B
d5_hPGCLC vs c42_DND1-
d5_hPGCLC vs c42_DND1+
Up: 1423
Down: 937
Up: 852
Down: 673
-Log10(Adjusted P-value)
Log2 Fold Change
Not significant
Up−regulated
Down−regulated
C
Log2(CPM+1)
d5_hPGCLC
c42_DND1−
c42_DND1+
D
SMARCA2
ETV5
BRCA2
BNC1
SFMBT1
FAM9C
BRDT
PROK2
PYGO1
SYCP2
SPEF2
DEFB1
STK33
DNAH1
PIWIL1
H1−1
TDRD1
TERB2
PIWIL2
CCDC62
CFAP91
SPATA31A7
DMRT1
SPATA31A1
SPATA31C1
SPATA31A3
RFX2
CFAP57
SERPINA5
SPATA31E1
SPATA31C2
SLC26A8
GARIN1B
MAS1
SEPTIN4
Log2(CPM+1)
d5_hPGCLC_1
d5_hPGCLC_2
d5_hPGCLC_3
c42_DND1−_1
c42_DND1−_2
c42_DND1−_3
c42_DND1+_1
c42_DND1+_2
c42_DND1+_3

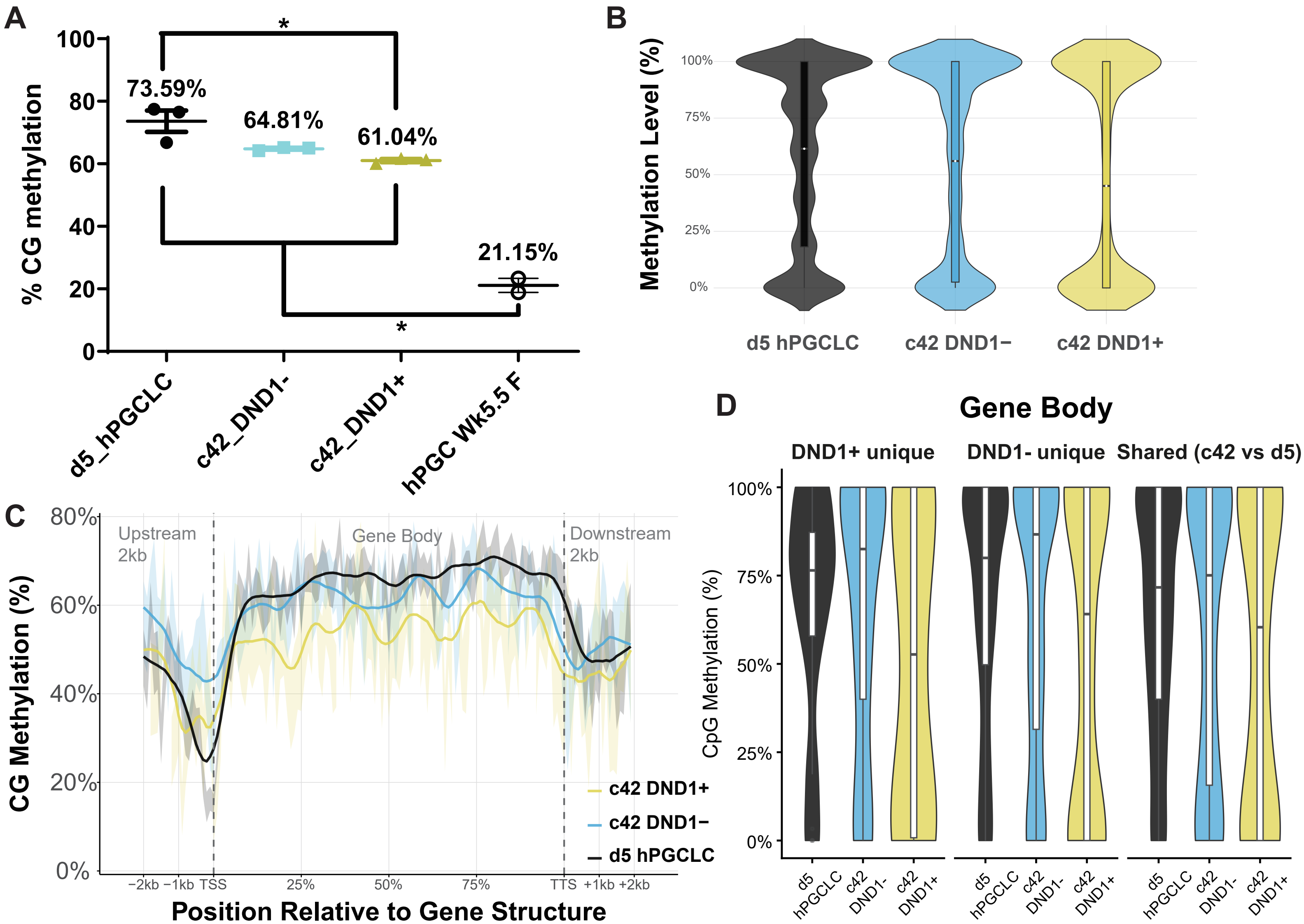

A
% CG methylation
100
80
60
40
20
0
73.59%
64.81%
61.04%
21.15%
*
*
d5_hPGCLC
c42_DND1-
c42_DND1+
hPGC Wk5.5 F
B
Methylation Level (%)
100%
75%
50%
25%
0%
d5 hPGCLC
c42 DND1−
c42 DND1+
C
CG Methylation (%)
80%
60%
40%
20%
0%
Upstream 2kb
Gene Body
Downstream 2kb
c42 DND1+
c42 DND1−
d5 hPGCLC
−2kb −1kb TSS
25%
50%
75%
TTS +1kb +2kb
Position Relative to Gene Structure
D
Gene Body
DND1+ unique
DND1- unique
Shared (c42 vs d5)
CpG Methylation (%)
100%
75%
50%
25%
0%
d5 hPGCLC
c42 DND1-
c42 DND1+

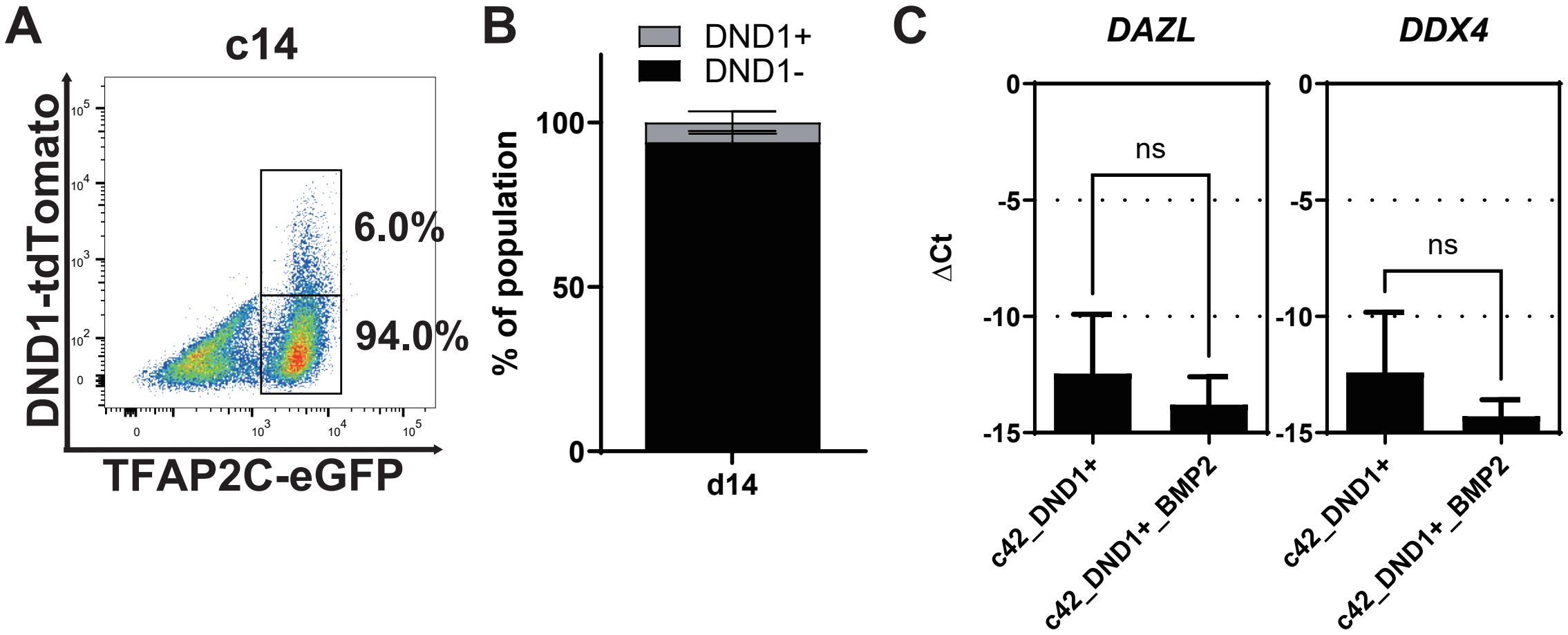
A
c14
DND1-tdTomato
TFAP2C-eGFP
6.0%
94.0%
B
DND1+
DND1-
% of population
100
50
0
d14
C
DAZL
DDX4
ΔCt
0
-5
-10
-15
ns
c42_DND1+
c42_DND1+_BMP2
