# Supplementary material for: Human primordial germ cell heterogeneity in vitro is associated with distinctive biological states
Source: Cell Mol Biol Lett. 2026 Jun 24;31:142. doi: 10.1186/s11658-026-00986-w (PMC13560305; doi:10.1186/s11658-026-00986-w)
Supplement: Supplementary file 2 [file 11658_2026_986_MOESM8_ESM.docx]

**Source Data 1. Full uncropped Gels for Figure S2C.**
